# How Structured Metadata Acquisition Contributes to the Reproducibility of Nanosafety Studies: Evaluation by a Round-Robin Test

**DOI:** 10.3390/nano12071053

**Published:** 2022-03-24

**Authors:** Linda Elberskirch, Adriana Sofranko, Julia Liebing, Norbert Riefler, Kunigunde Binder, Christian Bonatto Minella, Matthias Razum, Lutz Mädler, Klaus Unfried, Roel P. F. Schins, Annette Kraegeloh, Christoph van Thriel

**Affiliations:** 1 INM—Leibniz Institute for New Materials, Campus D2 2, 66123 Saarbrücken, Germany; linda.elberskirch@leibniz-inm.de; 2 IUF—Leibniz Research Institute for Environmental Medicine, Auf’m Hennekamp 50, 40225 Düsseldorf, Germany; adriana.sofranko@iuf-duesseldorf.de (A.S.); klaus.unfried@iuf-duesseldorf.de (K.U.); roel.schins@iuf-duesseldorf.de (R.P.F.S.); 3 IfADo—Leibniz Research Centre for Working Environment and Human Factors, Ardeystraße 67, 44139 Dortmund, Germany; liebing@ifado.de; 4 IWT—Leibniz-Institut für Werkstofforientierte Technologien, Badgasteiner Str. 3, 28359 Bremen, Germany; riefler@iwt.uni-bremen.de (N.R.); lmaedler@iwt.uni-bremen.de (L.M.); 5 FIZ Karlsruhe—Leibniz Institute for Information Infrastructure, Hermann-von-Helmholtz-Platz 1, 76133 Eggenstein-Leopoldshafen, Germany; kunigunde.binder@fiz-karlsruhe.de (K.B.); christian.bonatto-minella@fiz-karlsruhe.de (C.B.M.); matthias.razum@fiz-karlsruhe.de (M.R.)

**Keywords:** interlaboratory comparison, minimal information, quality criteria, description standards

## Abstract

It has been widely recognized that nanosafety studies are limited in reproducibility, caused by missing or inadequate information and data gaps. Reliable and comprehensive studies should be performed supported by standards or guidelines, which need to be harmonized and usable for the multidisciplinary field of nanosafety research. The previously described minimal information table (MIT), based on existing standards or guidelines, represents one approach towards harmonization. Here, we demonstrate the applicability and advantages of the MIT by a round-robin test. Its modular structure enables describing individual studies comprehensively by a combination of various relevant aspects. Three laboratories conducted a WST-1 cell viability assay using A549 cells to analyze the effects of the reference nanomaterials NM101 and NM110 according to predefined (S)OPs. The MIT contains relevant and defined descriptive information and quality criteria and thus supported the implementation of the round-robin test from planning, investigation to analysis and data interpretation. As a result, we could identify sources of variability and justify deviating results attributed to differences in specific procedures. Consequently, the use of the MIT contributes to the acquisition of reliable and comprehensive datasets and therefore improves the significance and reusability of nanosafety studies.

## 1. Introduction

Nanosafety studies are necessary for the assessment of the potential human health hazards of engineered nanomaterials (ENMs) and play a key role in addressing safety issues already during the development and design phase of new ENMs [1,2]. In vitro assays are used to assess the cellular effects of nanomaterials within decision-making frameworks [3]. At this level, mechanism-linked bioactivity assays, e.g., focusing on inflammatory responses, in combination with traditional cytotoxicity assays are thought to be important tools as long as they provide reliable and reproducible data [4,5]. These assays are easy to handle, cost-efficient, fast, and enable high-throughput screening [6,7]. Moreover, cells and in vitro systems derived from relevant target organs are available, according to the predominant exposure routes. Their use in regulatory processes is twofold as they can (1) be used to prioritize the generation of in vivo data and (2) facilitate the use of read-across approaches to avoid more animal experiments [8].

According to the advantages described above, the generated results are expected to provide comparability among laboratories and high confidence, which are required for safety assessment. In reality, established in vitro screening assays often lead to conflicting results, even if similar assays are used for the analysis of identical ENMs. Such contradictory and inconclusive research results have generated a wide discussion on data quality in nanosafety assessment [9,10]. This is clearly demonstrated by round-robin tests or interlaboratory comparisons, which are implemented in order to evaluate the comparability of results from multiple laboratories. To enhance the consistency in terms of quality and comprehensibility, standard operating procedures (SOPs) providing detailed instructions on how to perform different steps of the experiment are recommended. As an outcome of previous interlaboratory comparisons, the use of identical laboratory equipment, materials, and preceding training for the experimenters to minimize potential variability have been proposed [5,11]. This strategy improves the comprehensibility of results within single projects or collaborating laboratories [12]. However, considering the number of research groups in the field of nanosafety assessment, this time- and labor-intense approach cannot be realized throughout the community. Furthermore, the development of new approach methodologies (NAMs) as non-animal alternative models to mimic more realistic exposure scenarios and physiological conditions poses additional requirements due to their complexity [13,14]. These could only be partially described by already established toxicological assays, which is challenging for interlaboratory comparisons. Therefore, description standards and quality criteria for nanosafety studies are a substantial issue in academic and industrial research.

Various scientific projects and international organizations are developing standards or guidelines and promote harmonization throughout the nanosafety community [5,9,12]. Widely known examples of scientific guidelines directly related to nanosafety research are the Minimum Information Reporting in Bio–nano Experimental Literature (MIRIBEL) [15] and the documents of the Organization for Economic Cooperation and Development (OECD) Working party on Manufactured Nanomaterials (WPMNs) [16]. MIRIBEL focuses on minimum information that should be reported with study results of experiments investigating bio–nano interactions. The OECD WPMN works on the development of methods and strategies to identify and manage the potential health and environmental risks of nanomaterials and provide Test Guidelines (TGs) and Guidance Documents (GDs) [16]. In regulatory toxicology, which also involves industry and contract research, good laboratory practice (GLP) has been introduced as a comprehensive tool to ensure quality standards [17]. Even though in academia, this standard is not adhered to, it was recently shown that in nanoparticle-induced cytotoxicity testing, GLP can improve the quality of results from in vitro toxicity assays [5]. Following the recommendations of minimum information standards or guidelines will enhance the quality, reliability, and comprehensibility of published research data. Furthermore, the resulting published research data are limited in their findability, which complicates re-use. In this context, the collection of information about, e.g., experimenter-dependent handling or deviations from SOPs could support machine-readable data and metadata and therefore enhance findability and accessibility. To implement this information and achieve FAIRification of nanosafety research data, metadata schemas could be used [18]. Metadata schemas represent a common consensus on the hierarchical and relational structure of the descriptive information and specify designations of parameters and required content. A starting point for the development of metadata schemas is the previously introduced minimum information table (MIT), which is a collection of descriptive information and quality criteria based on existing standards or guidelines [19]. In order to facilitate multidisciplinary use, the MIT is divided into six modules: general information, material information, biological model information, exposure information, endpoint readout information, an, analysis and statistics. The relevance of containing descriptive information and quality criteria depend on the types of studies (e.g., in vitro or in vivo) or methods (e.g., dynamic light scattering or fluorescence microscopy) that have been used and therefore can be chosen in accordance with the individual requirements. Furthermore, the modular structure enables the addition of study- and method-specific parameters, e.g., there are multiple methods for ENM production or sample preparation that influence the behavior and interaction of ENMs in biological systems. The use of the MIT could support data collection throughout the data life cycle, bring improvement of data quality including completeness and reproducibility, and facilitate the data comprehensibility and re-usability of future research in accordance with the FAIR data principles.

**Figure 1:**
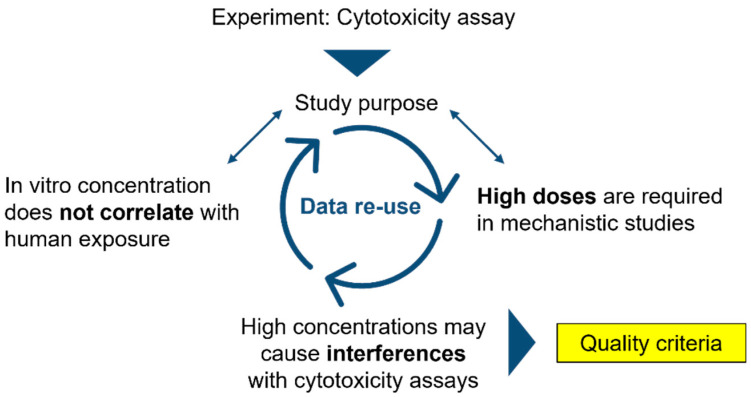
Role of descriptive information and quality criteria. Bold tags are used for highlighting keywords.

This study was designed to evaluate the parameters and criteria collected by the MIT to identify similar outcomes and explain differences in assay results based on the descriptive data and implementation of quality criteria. Descriptive information, provided by subject-specific metadata and quality criteria, plays a key role in data reliability and comprehensibility. Furthermore, descriptive information helps to understand the study purpose and design. The corresponding quality criteria are used to verify and evaluate the test system and study conditions. For example, the conduction of a cytotoxicity assay by using high ENM doses may not correlate with human exposure; however, high doses are required in mechanistic studies, which could be justified by the study purpose. In this context, quality criteria help to identify the interferences of ENMs with cytotoxicity assays, e.g., caused by the optical properties of the ENMs, and the adsorption or reactions with assay reagents or biomolecules and could improve the reusability and significance of the resulting data [20,21] (Figure 1).

The objective was to show how the content of the MIT could help to elucidate the variation in results to systematically understand the potential sources of variability in the study. Therefore, we demonstrate the study-dependent implementation and application of the previously defined quality criteria and minimum information by the implementation of a three-laboratory comparison of a nanotoxicity assay. The JRC reference nanomaterials NM101 and NM110 were chosen as ENM models for analyzing their effects on the human cell line A549. The study was conducted based on a round-robin approach that was performed in the framework of the EU FP7 nanosafety project ENPRA [22]. To be able to compare the experiments among laboratories, SOPs with detailed information and defined quality criteria on ENM handling and dispersion, cell cultivation and exposure, and the implementation of the WST-1 water-soluble tetrazolium salt-based assay were exchanged. The corresponding results were analyzed, and important aspects, deviations, and their effects were identified based on the MIT. Consequently, we were able to identify and justify reasons for the differences of the study results, which were mainly attributed to the MIT modules’ biological model information and exposure information.

## 2. Materials and Methods

### 2.1. Study Design and SOP Development

The study design phase started with the planning of the experiments and the acquisition of information on the used materials and methods and the roles of the various partners in the round-robin test. These steps were supported by the MIT groups “General Information”, “Material Information”, “Biological Model Information”, and “Exposure Information”. The experimental procedure was based on protocols previously used as a part of the ENPRA project [22,23]. Based on these protocols, (S)OPs for cell cultivation, probe sonicator calibration, sample preparation, exposure of the cells, and the WST-1 assay were defined. The SOPs were reviewed and discussed by one experimenter of each participating partner, whereby a device- and experience-independent description should be achieved. Additionally, the definition of the acceptance criteria (Table 1) served to ensure the comparability of the results despite the use of different materials and equipment. The detailed SOPs are available in the Supplement (Supplementary SOPs: SOP S1: Culturing A549 cells; SOP S2: Sample preparation; SOP S3: Sonicator calibration; SOP S4: Cellular viability—WST-1 assay in A549 cells). The procedures are briefly described below. Some materials and methods, e.g., sources of chemicals and instruments, instrument settings, cell culture conditions, etc., varied depending on the partner, but were recorded (Table S1: Minimal information table (MIT)).

### 2.2. Preparation of Test Materials

Test materials: Two test materials (JRC reference nanomaterials) were used that were also analyzed in the previously conducted ENPRA project [22]: NM 101 (Hombikat UV100; titanium dioxide (TiO_2_), rutile with minor anatase; mean diameter 29.9 nm ± 13.1 nm) and NM 110 (BASF Z-Cote; nonfunctionalized zinc oxide (ZnO), mean diameter 62.7 nm ± 32.6 nm) [24,25]. To exclude the effects of different material batches, one batch of each of these ENMs was split among the partners.

Sample preparation: The ENMs were supplied as dry powders. In order to obtain a concentration of 2.56 mg ENM/mL, ENMs were weighed into a 50 mL Falcon tube, followed by the addition of an appropriate amount of ultrapure water supplemented with 2% (*v*/*v*) fetal calf serum (2% FCS). Dispersion was achieved by ultrasonic treatment according to the ENPRA protocol: After application of a total energy of 7056 ± 103 J using a probe sonicator, samples were immediately transferred to ice (see Figure S1) and used within 1 h. The ENM stock dispersion was further diluted 1:10 in full-cell culture media without phenol red to obtain a concentration of 256 µg/mL (corresponding to 80 µg/cm^2^). Starting from this concentration, twofold serial dilutions were prepared in a mixture containing assay medium (cell culture medium with 10% FCS, *v*/*v*) and ENM dispersion medium (2% FCS in ultrapure water, *v*/*v*) at a ratio of 9:1 (*v*/*v*).

Probe sonicator calibration: The sonicators available at the three partner institutes were calibrated to obtain comparable ENM dispersions. The calibration procedure (see Supplementary SOPs) used was based on a calorimetric protocol described by Taurozzi et al. [26], which was further improved in the frame of the NANoREG project (NANoREG D4.12 SOP Probe Sonicator Calibration for ecotoxicological testing [27]).

According to this protocol, the sonicator was operated in continuous mode by starting with the lowest output setting and increasing it to 20% of the maximum amplitude. The temperature increase was recorded with a time resolution of no more than 30 s. The recorded data were plotted, and the temperature vs. time values were fit using a least-squares regression. The effective delivered power was calculated by the equation:

(1) $$P_{ac}\;\left(Watt\right)=\frac{\Delta T}{\Delta t}\;MC_{P}$$

where *P_ac_* is the delivered acoustic power (W), ∆*T*/∆*t* the slope of the regression curve with temperature T (K) and time t (s), *C_P_* the specific heat of the liquid (4.18 J/g × K for water), and M the mass of the liquid (g). To deliver a total energy of 7056 ± 103 J, the amplitude settings and the sonication time t were adjusted according to the equation [28]:

(2) $$t\;\left(s\right)=\frac{E\;\left(7056\;J\right)}{P_{ac}\left(W\right)}$$

### 2.3. Sedimentation Analysis

Cell responses to ENM exposure are considered to be dependent on the ENM dose delivered to the cells rather than due to the administered concentration during an in vitro experiment [18]. ENM transport in liquid media is determined by diffusion and gravitational settling and therefore dependent on ENM size (including aggregates), density, and agglomeration behavior [29]. The resulting particle sedimentation was expected to influence the particle delivery during the exposure phase. In addition, sedimentation might affect the quantitative handling of ENMs already during sample preparation, thereby influencing the effective administered ENM concentration. In order to determine the relevance of particle sedimentation for both instances, the time-dependent sedimentation of the used ENMs dispersed in cell culture medium was examined by use of UV-Vis spectroscopy and compared to theoretical sedimentation curves, described in the Supplement (Method S1). Measurements were performed with a UV-Vis spectrometer ( UV-2600, Shimadzu Deutschland GmbH, Duisburg, Germany) at room temperature (20 °C within a laboratory with air conditioning), using a cuvette holder. In a first step, spectra of the cell culture medium were recorded as a reference. After cleaning and drying, the cuvette was refilled with the ENM dispersions prepared as described in Section 2.2. By adjusting the time interval between measurements, long-term studies over up to 45 h were performed. At high particle concentrations (c_0_ = 2.56 mg/mL), a 2 mm (layer thickness) quartz cuvette was used, while all measurements at lower particle concentrations were performed with standard (10 mm) polystyrene cuvettes.

### 2.4. Cell Culture

The cell line A549 (DSMZ No.: ACC 107) [30] was used as a model for human alveolar epithelial type II cells as it is widely used for nanotoxicity studies [31]. Cells of the same passage were distributed among the three partners. A549 cells were cultivated in Dulbecco’s Modified Eagle’s Medium (DMEM) supplemented with 10% FCS in 75 cm^2^ cell culture flasks in a humidified atmosphere at 37 °C and 5–10% CO_2_ (constant value, depending on the sodium bicarbonate concentration of the used medium). At a confluence of 70–90%, cells were detached by the addition of 2 mL 0.05% Trypsin/0.02% EDTA, incubation at 37 °C for 3–5 min, and centrifugation at 200× *g* for 5 min. For subcultivation, cells were replated at a ratio of 1:5–1:10 into 75 cm^2^ cell culture flasks.

### 2.5. Exposure of Cells to ENMs

For cytotoxicity experiments, A549 cells were seeded into 96-well plates at a density of 1 × 10^4^ cells per well and allowed to attach for 24 h. Cells were then washed using serum containing medium without phenol red. Then, 100 µL of freshly prepared ENM dispersions with administered concentrations of 0.3125 µg/cm², 0.625 µg/cm², 1.25 µg/cm², 2.5 µg/cm², 5 µg/cm², 10 µg/cm², 20 µg/cm², 40 µg/cm², and 80 µg/cm² (see Section 2.2) or medium (as the control representing viable cells) was added and the cells incubated for 24 h. Afterwards, two of all replicates were exposed to 0.5% Triton-X 100 for additional 15 min at 37 °C (as the cytotoxicity control).

### 2.6. Viability (WST-1 Assay)

To determine viability (metabolic activity), the WST-1 assay (Roche Diagnostics) was used according to a protocol initially adapted by Vietti et al. [32] and further adjusted in the frame of the ENPRA project [22]. After incubation with 10 µL of WST-1 solution for 1 h, absorption was measured at 450 nm and at 630 nm as the reference wavelength.

### 2.7. Analysis and Statistics

The WST-1 assay was performed by each experimenter (all in all, 6) within the three partner institutes (nested design) three times independently (*n* = 3, biological replicates) on different days, using different cell passages, freshly dispersed ENMs, and freshly diluted Triton-X-100. Four wells per treatment per setup were used and served as technical replicates. All these different “factors” were used as possible sources of variance in the statistical analysis. For data analysis, the raw absorption data were entered into a shared MS Excel calculation template. Corrected absorption values and normalized values were calculated as follows:

1. Corrected absorption: To correct for unspecific medium and ENM absorption, the absorption of the medium and the ENM-containing (at the corresponding concentrations) medium at 450 nm was subtracted from the corresponding absorption values obtained in the presence of cells. The values at 450 nm were further corrected by subtraction of the reference absorption values obtained at 630 nm. To further correct for cell absorption (see SOP), the absorption of cells treated with the positive control (0.5% Triton X-100) was subtracted from the absorption values of the ENM-treated cells;
2. Normalized values: The corrected quadruple ENM-treated cell sample values from each treatment group were converted to normalized values by the following equation and used for further statistical analyses:
  (3) $$normalized\;value\;x\;\left(\%\right)=\frac{A\;\mathrm{sample}}{A\;\mathrm{untreated}\;\mathrm{control}\;}\times 100$$

The statistical analyses were performed using IBM SPSS Statistics for Macintosh, Version 28.0 (IBM Corp. Released 2021. IBM SPSS Statistics for Macintosh, Version 28.0. Armonk, NY, USA: IBM Corp) and GraphPad Prism version 9.2.0 for Mac OS X (GraphPad Software, San Diego, CA, USA: www.graphpad.com, accessed on 20 February 2022). In general, two statistical approaches were used to explore the data, one approach with and another without normalization to the respective control conditions. The normalized data were expressed as the percentage of the controls (range: 0% to 100%), and these values were used to calculate sigmoidal functions describing the inhibitory potency of the tested concentrations. The half maximal inhibitory concentrations (IC_50_) for the separate datasets generated by each experimenter were calculated based on normalized values of the three biological replicates. Before calculating these values, the data were inspected for outliers (see Krebs et al., 2019 [33]) using the algorithm implemented in the box and whiskers plot procedure of SPSS. Here, outliers are defined as values above the 3rd quartile + 1.5 × interquartile range (IQR) or below the 1st quartile—1.5 × IQR. This procedure does not assume normally distributed values and is therefore suitable for cytotoxicity tests where this assumption is difficult to achieve due to the often skewed distribution of the dependent variable in the range of the high or low concentrations. To avoid non-linear transformation introduced by the normalization to the control wells, we used the corrected absorption as provided by the plate readers available at the partners labs for the second analysis. Again, these data were inspected for outliers as described before. To identify factors contributing to the interlaboratory variation as nested (hierarchical), an analysis of variance (ANOVA) model was used. The model (UNIANOVA in SPSS 28.0) consisted of four factors that were (1) concentration (fixed factor), (2) biological replicate (random factor), (3) partner (random factor), and (4) experimenter (nested factor within partner). All possible main effects and interactions were included in the model, and post hoc tests and paired comparisons were used to explore the significant effects in detail.

The IC50 values and their respective 95% confidence intervals (95%-CI) were calculated in Prism using the nonlinear fitting algorithm “[Inhibitor] vs. normalized response—Variable slope” that uses the following equation to describe the dose–response relationship obtained by the six experimenters on the three biological replicates with the concentration (*Y*) and the normalized response in the WST-1 assay (*X*).

(4) $$Y=\frac{100}{1+{\left(\frac{IC_{50}}{X}\right)}^{HillSlope}}$$

## 3. Results

### 3.1. Cytotoxicity

The WST-1 assay, indicating metabolic activity, was selected to analyze the effects of NM101 and NM110 on cell viability after 24 h of exposure. The round-robin testing was carried out by two experimenters each at the three involved institutes.

**Figure 2:**
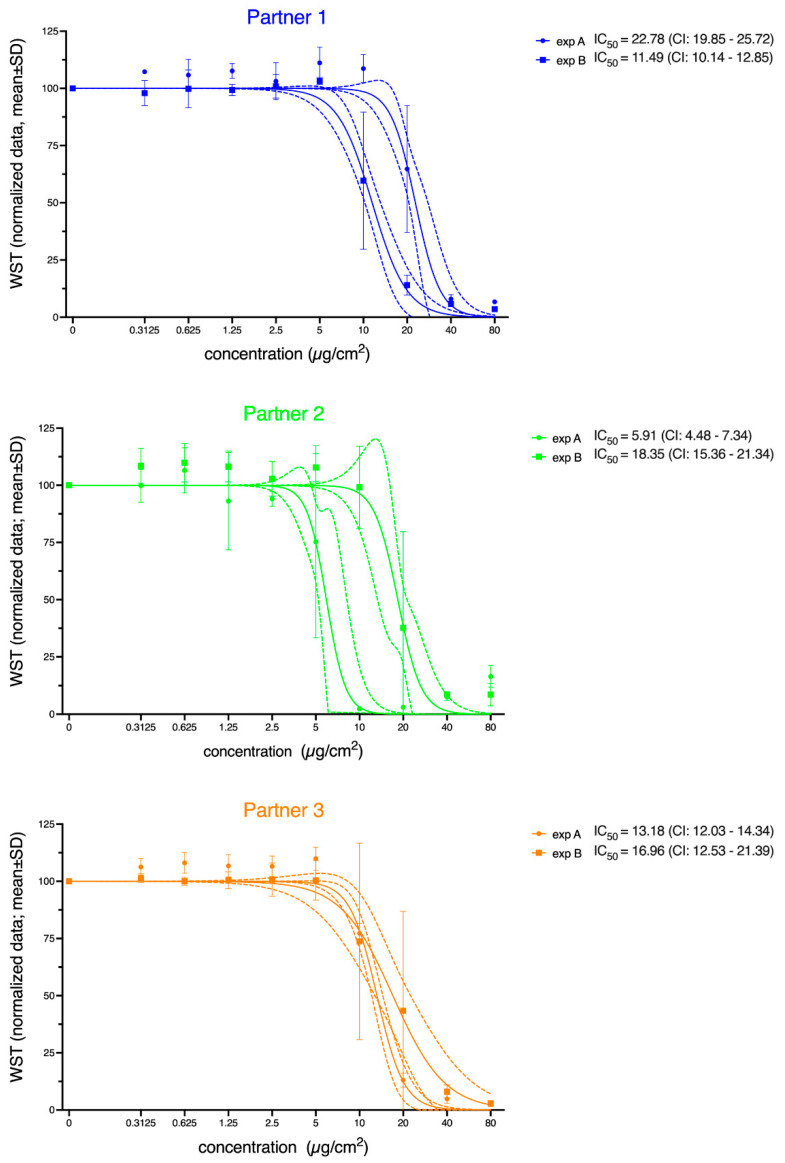
Viability of A549 cells, as indicated by the WST-1 assay after 24 h of exposure to NM110 (ZnO). Normalized data are presented corresponding to measurements performed by the various experimenters (exp A/B) at the participating institutes (Partners 1–3). Solid lines represent the nonlinear regression based on the calculated mean values; dashed lines represent the corresponding 95% confidence bands. The calculated IC_50_ values are given in the figure legends along with the corresponding confidence intervals (CIs). The concentration is the administered concentration of the ENMs based on the initial dispersion.

As shown in Figure 2, all experimenters demonstrated a concentration-dependent decrease in the viability of A549 cells down to 0% after exposure to NM110 (ZnO). For NM101 (TiO_2_), no concentration-dependent decrease of the viability was observed (F_(9,710)_ = 1.22; *p* = 0.28; Supplementary Figure S1). Therefore, only the results of NM110 were used here to evaluate whether the MIT can be used to identify sources of variance.

As described in Section 2.7, outliers were excluded before estimating the IC_50_ values mathematically. The percentage of outliers in the normalized dataset for the six experimenters ranged from 0% to 4.1%. Thus, a maximum of 5 of the 120 values (4 [measures/ well] × 3 [biological replicates] × 10 [concentrations]) that were obtained in the experiments of one experimenter were recognized as outliers. The results of the nonlinear fitting algorithm for the outlier-corrected, normalized data are shown in Figure 2. The three partners and the two experimenters within these institutes are given separately. The normalized data clearly showed variations in the IC_50_ values obtained by the six experimenters (Figure 2). The IC_50_ values varied in the range of 5.91–22.78 µg/cm^2^. Moreover, the 95% confidence intervals (CIs) indicated differences in the accuracy of the IC_50_ estimates across the experimenters. Experimenter B at Partner 1 and Experimenter A at Partner 3 obtained very narrow 95%-CIs, indicating high reproducibility across the technical and biological replicates, while other sets spanned a broader range of uncertainty. Figure 2 also illustrates that the dose–response curves of the two experimenters at Partners 1 and 2 ran parallel to each other, while at Partner 3, the slopes of the curves differed, and they crossed each other at 10 µg/cm^2^.

To explore these differences in more detail, as the next step of the analyses, the sources of these variations should be identified by using the nested (hierarchical) ANOVA model. Explorative data analyses and box and whiskers plots were used to identify outliers per concentration (see the Supplement) within the single datasets of the six experimenters. In the datasets of three experimenters, no outliers were detected. In the other three sets, only a few outliers were identified (0.8%, 1.7%, and 2.5% of the 120 data points/experimenter). For these sets, mean values were calculated omitting the outlier values and subsequently used to substitute the outlier values. Thereby, the calculation of the sum of squares (total variation observed in the sample/factor of the model) and the mean square (total variation divided by the degrees of freedom) given in the ANOVA table were based on an identical number of observations. The results of the ANOVA are given in Table 2.

**Figure 3:**
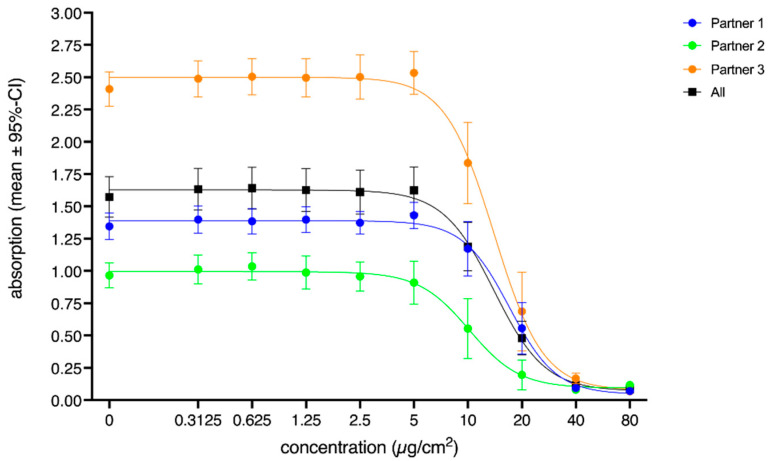
Concentration-dependent effects of the random factor partner on the absorption. Values represent mean values (corrected absorption) of the three partners, as well as the average (black square). Error bars indicate the corresponding 95% confidence intervals. The concentration is the administered concentration of the ENMs based on the initial dispersion.

First, the main effect of the fixed factor concentration was shown to be highly significant (F = 11.75, *p* < 0.001; see the black squares in Figure 3). This strong effect of the applied concentrations on the corresponding corrected absorption values of the three partners is illustrated by the roughly sigmoidal courses (see Figure 2 for plots using normalized data) of the curves, when plotted against concentration. The curves approach y = 0 only at the highest concentrations. The significant main effect of the random factor partner (F = 8.99, *p* = 0.02) is also visible in Figure 3 as Partner 3 yielded an approximately two-fold higher absorption value than the other two partners. However, as indicated by the significant interaction of the factors partners and concentration (F = 6.96, *p* < 0.001), these absorption differences were more pronounced at the lower concentrations. At the two highest concentrations, the absorption values of all partners approached zero. For all partners, post hoc tests revealed that the absorption values obtained at 10 µg/cm^2^ differed significantly from the values obtained at the next lower concentration (Partner 1: mean difference: −0.26, *p* < 0.001; Partner 2: mean difference: −0.36, *p* < 0.001; Partner 3: mean difference: −0.70, *p* < 0.001). This is also the concentration where the Dunnett t post hoc test of the random factor concentration indicated the first significant difference from the control condition (mean difference: −0.39, *p* < 0.001). However, from concentration step 20 µg/cm^2^ on, no significant differences in the measured absorption (see overlapping CIs) among the partners could be found. The functions plotted in Figure 3 also illustrate the significant interaction of the two factors: partner and concentration. The slopes of the sigmoidal curves were slightly different, while Partner 2 yielded a flatter slope, while the slope of Partner 3 was steeper.

By evaluating the records of the experimenters in the MIT, we could identify deviations in the seeded cell numbers (see MIT parameter “cell seeding details”) as a possible reason for the high absorption values of Partner 3 at low concentrations. According to the SOP, 10,000 cells per well in a 96-well plate format should be seeded for the WST-1 assay. The deviation of Partner 3 was caused by the attempt to meet the quality criteria of treating the cells at 70% confluence. Partner 3 noted that this was not achieved with the given cell number of 10,000 cells. Therefore, the information documented according to the MIT revealed that the cultivation conditions differed between the individual partners, resulting in variable cell growth.

**Figure 4:**
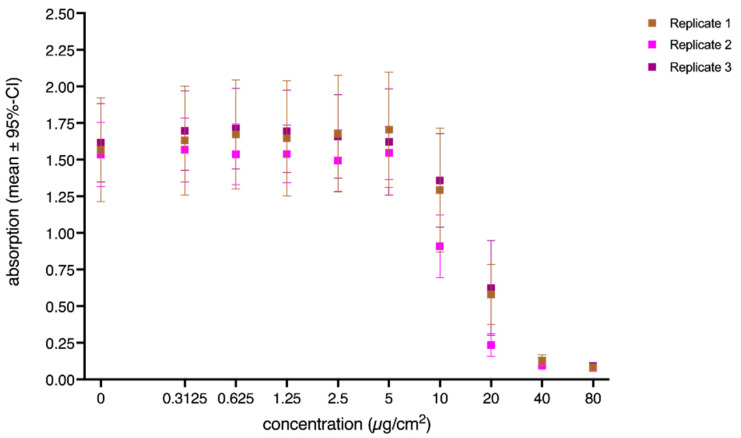
Concentration-dependent effects of the random factor replicate on the absorption. Values represent mean values (corrected absorption) of the three biological replicates. Error bars indicate the corresponding 95% confidence intervals. The concentration is the administered concentration of the ENMs based on the initial dispersion.

Interestingly, the random factor replicates yielded neither a significant main effect, nor a significant interaction with the fixed factor concentration (Table 2 and Figure 4) Thus, the replication of the experiments yielded comparable results (Figure 4) across the entire concentration range. Overall, Replicate 2 revealed slightly lower absorption values; however, in general, the results were in good agreement, and overall, the replication of the experiments showed comparable results.

**Figure 5:**
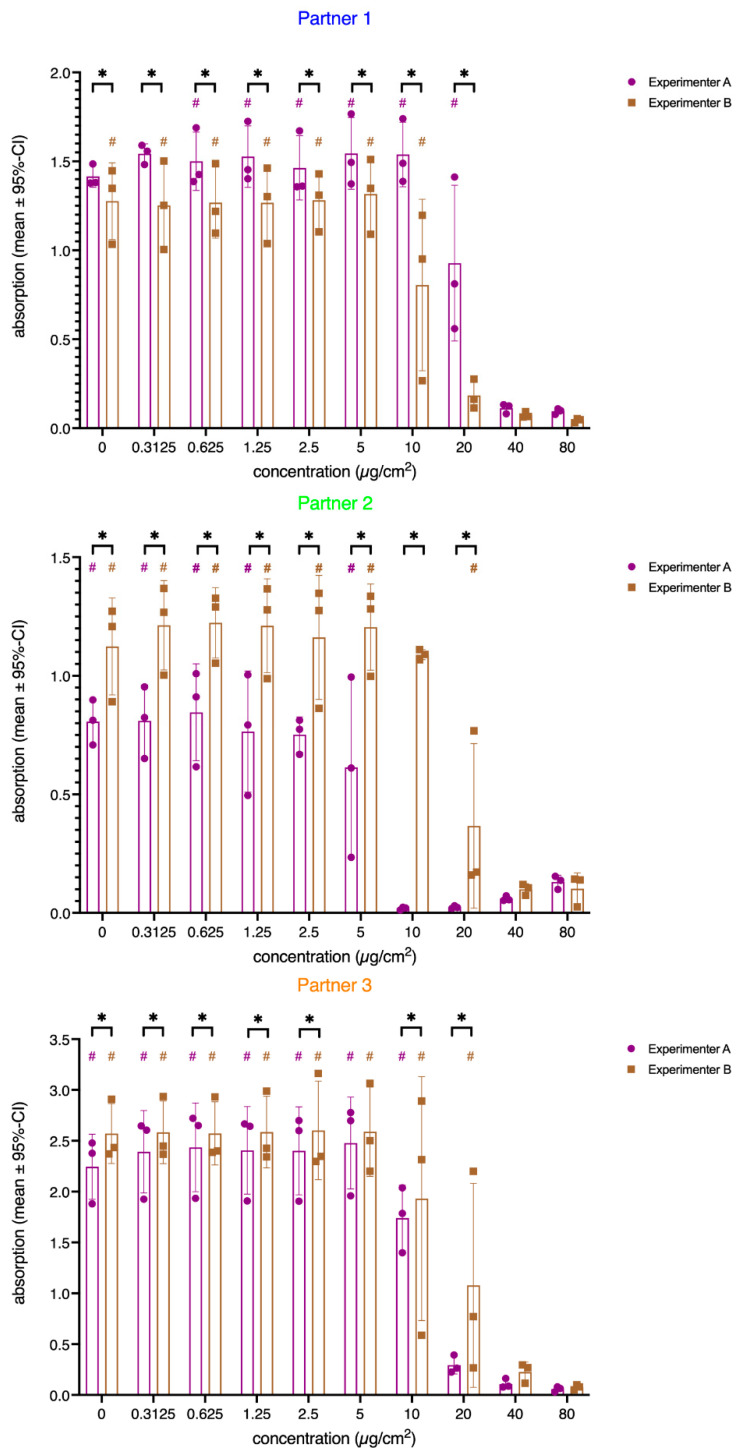
Bar chart showing the interaction of the factors concentration (x-axis), partner (panels Partner 1–3), experimenter within the partners (color-coded bars), and biological replicate (purple and brown dots and squares). Bars represent mean values (corrected absorption), and error bars indicate the corresponding standard deviation. Individual measures of the three biological replicates are given as color-coded dots and squares. # above the bars indicate significant differences among the biological replicates of the two experimenters at a certain concentration step (*p* < 0.05 according to pairwise comparisons, adjusted for multiple tests). Adjusted significant comparisons between the two experimenters within in the partners are given above the bars (*: *p* < 0.05 according to pairwise comparisons, adjusted for multiple tests). The concentration is the administered concentration of the ENMs based on the initial dispersion.

However, the ANOVA results (Table 2) revealed that within the three partners (nested effect), the values of the three replicate sets markedly differed for Experimenters A and B, especially at lower concentrations. Thus, a huge amount of the variation can be explained by differences between the three replicates performed by the two experimenters of the three partners. This highly significant interaction of all factors (F = 25.52, *p* < 0.001) involved in the model is depicted in Figure 5.

Within the three partners, the WST-1 results were differently affected by the concentration and/or the replicates of the respective experimenters. For all partners, the two involved experimenters produced significantly different results for most of the concentration steps. These differences were systematic, as one experimenter always yielded lower absorption values than the other at least up to the concentrations 2.5 µg/cm^2^ and 5 µg/cm^2^. Only at the lab of Partner 3, the two experimenters yielded comparable values for the 5 µg/cm^2^ concentration, a generally non-cytotoxic concentration (see the Dunnett t post hoc test). For Partners 1 and 2, the two experimenters yielded highly different results for the concentration steps 10 µg/cm^2^ and 20 µg/cm^2^. These differences were to some extent less pronounced for Partner 3. However, all paired comparisons were significant, and here, Experimenter B yielded highly significant differences among the biological replicates. Surprisingly, the cell viability increased with concentrations higher than 20 µg/cm^2^ for Experimenter A at Partner 2 with no strong variation across the replicates. The concentration-dependent differences of the replicates were less pronounced for Partner 3, but here, Experimenter B showed huge differences among the replicates. In general, the variation across the replicates was highest in the concentration steps around the IC_50_ values (see Figure 1). However, even in this dose range, some experimenters showed almost no (see Partner 2) or small differences (see Partner 3, Experimenter A).

Again, we matched these results to the entries in the MIT. Some explanations could be found in the group “Endpoint Read Out Information”. As an acceptance criterion for assessing the test system at its start, 70% confluence of the cells is defined in the SOP. It has to be mentioned that this value was estimated by the experimenters and no values were given by Partner 3. Nevertheless, the differences in absorption between the two experimenters of Partner 2 may be in part due to the fact that Experimenter A had only 40–50% cell confluence and Experimenter B between 70% and 80%. Lower cell numbers may result in decreased absorption in the WST-1 assay because of less reagent reduction. Likewise, Experimenter A of Partner 1 estimated 80–85% confluence and Experimenter B estimated values of 70–90%. This is reflected by the absorption values in Figure 5, revealing that the absorption values of Experimenter A were usually significantly higher compared to Experimenter B.

Additionally, if a lesser quantity of cells were treated, toxicity may also be increased at lower ENM concentrations, which may explain the difference in IC_50_ values between Experimenter A with an IC_50_ of 5.3 and B with an IC_50_ of 16.7. Kim et al. investigated the effect of cell density and nanoparticle uptake, and they showed that lower cell density results in higher nanoparticle uptake [34].

A further acceptance criterion was defined in the SOP for the test system at the end of compound exposure. At this time point, the absorption should be between 0.5 and 2 with a standard deviation of <0.3. The standard deviation criterion was met by all experimenters except for one concentration (2.5 µg/cm^2^) in the sets of Experimenter B of Partner 3, showing a standard deviation of 1.03. The criteria for raw absorption values were met for most of the experiments, except for Partner 3. Here, Experimenter A obtained absorption values between 2.2 and 2.5 in Replicate 1 and 2.4 and 2.6 in Replicate 3. Experimenter B measured absorption values between 2.8 and 3.0, 2.3 and 2.6, and 2.2 and 2.4 in Replicates 1–3, respectively. This could be due to the increased cell numbers seeded into the well plates. Although the measured values were outside the acceptance criteria, the resulting curves and the IC_50_ of Partner 3 were within a suitable range. Overall, the acceptance criteria for the test system were not achieved by all experimenters.

**Figure 6:**
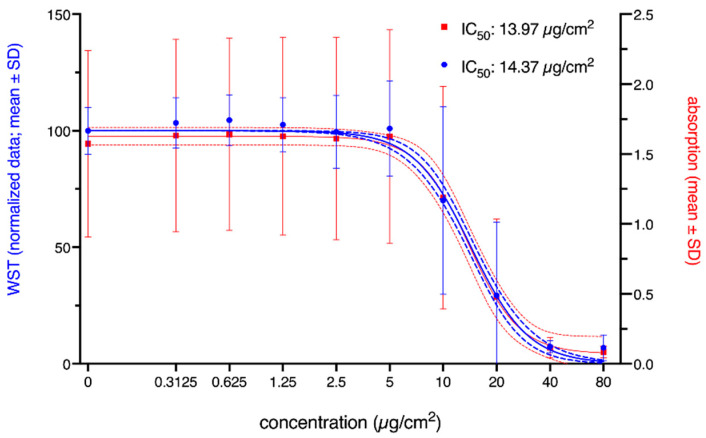
Viability of A549 cells, as indicated by the WST-1 assay, after 24 h of exposure to NM110 (ZnO). Both normalized data (blue) and corrected absorption (red) are given. Solid lines represent the nonlinear regression based on the calculated mean values; dashed and dotted lines represent the corresponding 95% confidence bands. The calculated IC_50_ values are given in the figure legend. The concentration is the administered concentration of the ENMs based on the initial dispersion.

Finally, the overall impact of normalization should be analyzed. Figure 6 shows the fitted sigmoidal curves for normalized and raw absorption data of the WST-1 assay. While the estimated IC_50_ values and fitted curves were almost identical, the confidence intervals, as well as the SDs of the raw data were wider. This is also expressed in the goodness-of-fit values with an R2 of 0.50 for the raw data and an R2 of 0.80 for the normalized data. Thus, by normalizing the data to the respective control condition, the impact of some sources of variance identified and described in the previous sections could be reduced.

### 3.2. ENM Sedimentation

**Figure 7:**
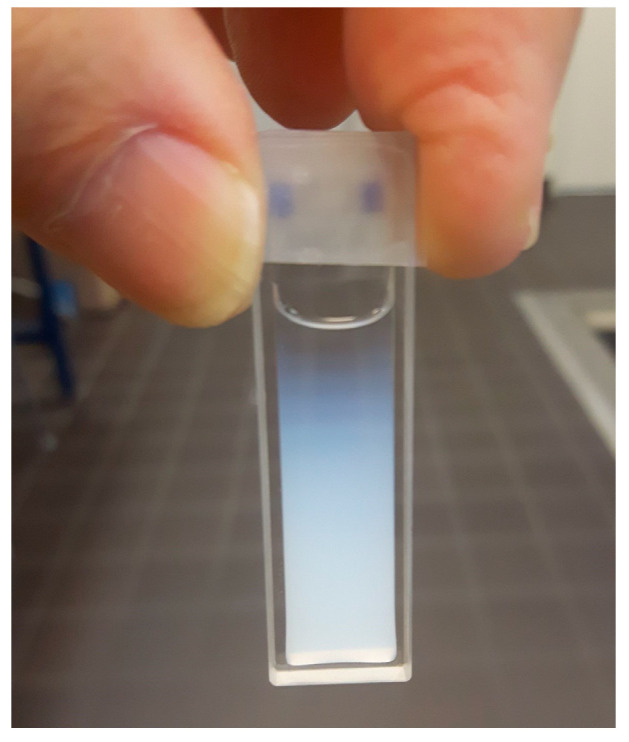
Sedimentation of ZnO (NM110) nanoparticles at c = 2.56 mg/mL and t = 30 h after dispersion in cell culture medium. In the UV-Vis spectrophotometer used for qualitative analysis, the light passes through the suspension as a flat vertical light sheet in the lower third of the cuvette.

Deviations in the absorption values at IC_50_ not only between partners, but also between the replicates of single experimenters could be due to the non-uniform dosage of the ENMs. Such inconsistent dosage might be caused by particle agglomeration and/or resulting sedimentation during sample preparation. In order to estimate the influence of these effects on the reproducibility of the assay, the stability of the ENM dispersion was tested by sedimentation analyses. Due to the unknown agglomeration state of the nanoparticles in complex media, e.g., cell culture media, theoretical considerations about the sedimentation (see the Supplement) cannot be used to predict particle sedimentation times. However, qualitative comparisons are possible. An example illustrating qualitative particle sedimentation analysis by recording UV-Vis spectra is shown in Figure 7 for the used ZnO (NM110) nanoparticles. A gradient of nanoparticles from the top to the ground of the cuvette is clearly visible.

**Figure 8:**
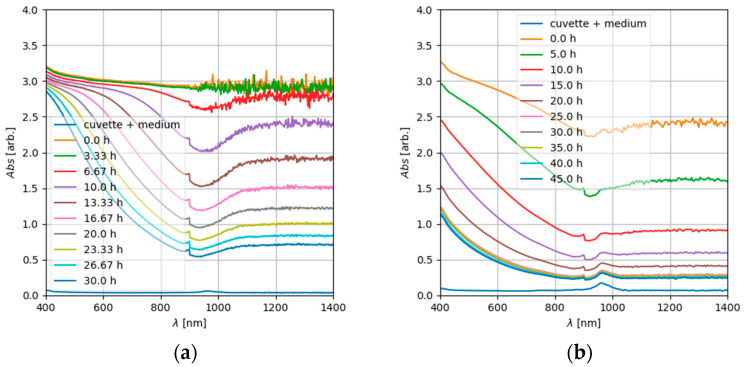
Time series of the UV-Vis spectra of NM110 TiO_2_ dispersed in cell culture medium (treatment; see Section 2.2) at a concentration of c_0_ = 2.56 mg/mL (**a**) and 0.256 mg/mL (**b**); the lowermost spectrum is from the cuvette and the medium without particles.

In the UV range, absorption was very strong and noisy and clipped at units higher than 10. Over the course of time, within a timescale of several hours, the absorption decreased significantly in the visible and infrared wavelength range (Figure 8). This can be explained by a reduction in the number of particles due to sedimentation. At particle concentrations used for cytotoxicity assays (e.g., c = 256 µg/mL, the highest concentration used), a comparable decrease in the absorption over time was observed (Figure 8, right). Only at the lower concentration range used for the cytotoxicity assays (e.g., c = 8 µg/mL, corresponding to 2.5 µg/cm^2^), hardly any decline in the absorption over time was observed. However, under the conditions used, the observed particle sedimentation and the concomitant increase in the delivered dose did not result in a decrease of cell viability.

**Figure 9:**
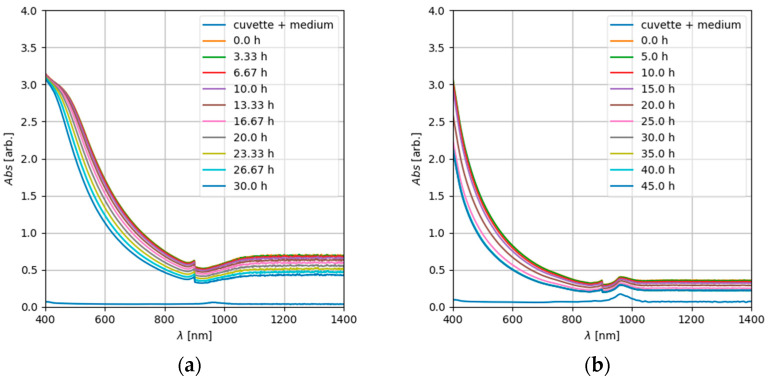
Time series of UV-Vis spectra of ZnO nanoparticles (NM 110) dispersed in cell culture medium (treatment; see Section 2.2) at a concentration of c = 2.56 mg/mL (**a**) and 0.256 mg/mL (**b**); the lowermost spectrum represents the medium without particles.

In comparison, the dispersion of NM 110 (ZnO) particles (Figure 9a for c_0_ = 2.56 mg/mL) exhibited a sloping spectrum in the visible wavelength range and a roughly constant, low absorption in the infrared range. Over time, only a minor decrease in the overall absorption was observed, indicating a limited alteration of the particle dispersion, which in the case of ZnO might be due to sedimentation, but also to particle dissolution [35]. However, apparently not all ZnO particles transformed during the time course of the experiment. In contrast, a prompt transformation of ZnO nanoparticles in the cell culture medium was reported by Ivask et al. [35].

The results indicated that due to the slowness of the sedimentation process (timescale of hours), a strong influence on quantitative and reproducible handling of the dispersion during sample preparation can be neglected. Following the SOP, the ENM dispersions should be utilized within 60 min.

In comparison, particle sedimentation during the exposure of submerged, static cell cultures increased the dose delivered to the cells over the time course of the experiment. Particle sedimentation might be increased by agglomeration but, on the other hand, decreased by dissolution. Dissolution is regarded to be relevant for the impact of ZnO nanoparticles. Data considering the sedimentation and dissolution behavior of ENMs should be provided (see [20], Supplementary File) A recent review on mathematical models including in vitro dosimetry was provided by Lamon et al., 2019 [36].

## 4. Discussion

Data from nanosafety studies frequently show discrepant outcomes and low reproducibility even within single studies, which leads to contradictory assessments and limits their use for regulatory purposes. In this round-robin test, we aimed to improve the comprehensibility of variable results by using the MIT containing descriptive information and quality criteria [19]. The round-robin test was composed by a WST-1 assay for toxicity measurement of ENMs to evaluate various sources of variance such as the laboratory (partners), the experimenters performing the assay, and the independent replicates of these experiments. Initially, SOPs for cell cultivation, ENM preparation, and WST-1 performance were developed. Furthermore, the SOPs included acceptance criteria to monitor the biological model system. Since the chosen test procedure is a common working process in a toxicology laboratory, we assumed that the implementation could be carried out by the experimenters without further training or explanation. In addition, it should be assumed that the use of different materials and devices, provided they are approved for the described application, should only have a limited influence on the results. In fact, this is not confirmed by various published interlaboratory comparisons [11,12,22]. In previous round-robin tests in the field of nanosafety research, the exchange of materials and the training of experimenters played a key role in achieving less variability in the study results [5]. In contrast, we used the MIT to identify reasons and sources of variances in the results. The main reasons for variances could be found in the MIT groups General Information, Biological Model Information, Exposure information, and Endpoint and Readout Information. Deviations from the SOP based on the experimenter’s decision were a main factor.

Two experimenters at each partner laboratory performed three rounds (replicates) of the test procedure. This setup allowed comparison of variances within one laboratory and between the laboratories concerning the WST-1 assay results. Variances could indeed be detected between the three partners, but also between the experimenters within the same laboratory. The results appeared to be influenced by the so-called human factor. Specific laboratory practices such as pipetting techniques, which are not explicitly described in the SOP, or experience and routine regarding the test procedure can be mentioned as a source of variance. Therefore, critical steps in the protocol need to be identified and defined in the SOP to ensure the overall robustness and reproducibility of assay results within and between different laboratories. Additionally, appropriate and standardized ENMs, including their reliable and curated characterization, are needed to ensure the test performance.

Based on the concentration-dependent results obtained for NM-110, the IC_50_ values were calculated. The values varied in the range of 5.91–22.78 µg/cm^2^. In comparison, a multilaboratory toxicological assessment by Kermanizadeh et al. [22] obtained IC_50_ values in the range of 5.39 ± 6.03 µg/cm^2^. Differences in these values were attributed to potential differences in the handling of ENMs or a variation in cell stocks. ENMs tend to age at different conditions during storage, such as, e.g., silver ENM [37]. A single-laboratory study by Thongkam et al. obtained a higher IC_50_ value of 44 µg/cm^2^ while using the same ENMs. Moreover, in the study of Thongkam et al., cells were seeded at lower densities in order to align the conditions to mutagenicity studies performed in this context. Overall, literature data on IC_50_ values for NM110 or ZnO varied in the range of 2.53–44 µg/cm^2^, which is comparable to the results of the presented round-robin test (Table 3).

### 4.1. Statistics

In risk assessment procedures, in vitro cytotoxicity assays are often used in the context of read-across approaches [41] as they provide, first, quantitative information about adverse effects. Thus, these estimations should be reliable and valid, and the data handling and statistical analyses should be appropriate and described in detail. By analyzing and describing the proportion of outliers using standardized statistical methods, first, information about the quality of the test results could be given and bias due to extreme values could be excluded. Our analysis showed that only a few values of the WST assay were identified as outliers, and they were either excluded from the analysis or substituted by mean values of the remaining measures. Our results also showed that some interlaboratory variation could be reduced by using normalized data (see Figure 6). However, these mathematical transformations mainly affect the confidence interval of the cytotoxicity estimations without having a strong impact on the absolute values. When providing in vitro data in repositories following the FAIR principles, information about outliers and normalization should be given to enable quality assessment of the provided information.

### 4.2. Metadata Acquisition

**Figure 10:**
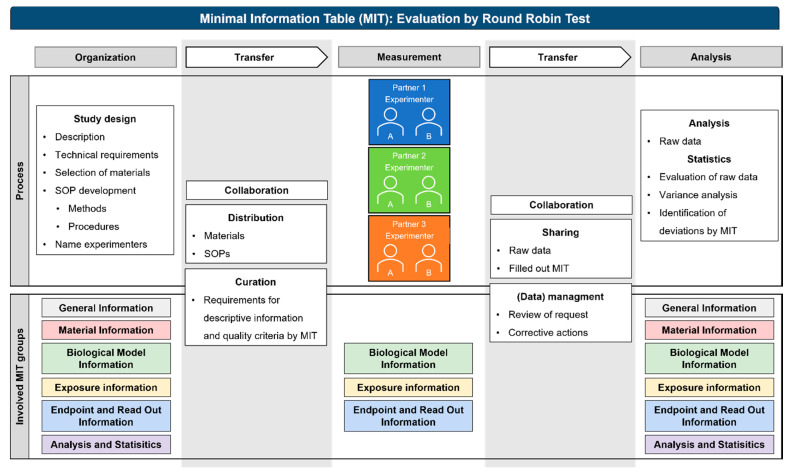
Evaluation process of the MIT by round-robin test. The planning and implementation of the round-robin test could be separated into organization, measurement, and analysis. The connective interfaces are transfer areas with collaboration processes including distribution, curation, sharing, and (data) management.

The round-robin test was performed to evaluate the applicability and contribution of the MIT to the analysis and identification of sources of variance. The process of the round-robin test comprised the organization of the study design, including the development of the SOPs, the measurements performed by the experimenters, up to the analysis of the results (Figure 10). Nevertheless, these are only major steps that need to be considered within a round-robin test. With regard to data comprehensibility and reuse, specifically, the interfaces of information and data transfer play a key role with regard to data comprehensibility and reuse. Usually, data are stored on private servers, and the data are published in the end. However, the researcher decides which data and information will be shared. In contrast, digital data transfer and non-publication outputs are demanding for collaboration, curation, and (data) management activities. This process includes recording important information about the data and their generation, sources, analysis methods, and changes to the data, which can be fulfilled by the defined requirements for descriptive information and quality criteria given in the MIT. Its use could result in an ongoing data curation throughout the research data life cycle, contributing to data preserving in, e.g., repositories and enabling access and reuse in the future.

However, there are aspects and actions that cannot be achieved by using the MIT as a single tool. Therefore, further developments regarding comprehensibility and re-use are necessary. For the first transfer point, it should be considered that, e.g., SOPs need to be available and access conditions should be regulated; furthermore, they should be provided with an identifier (e.g., DOI). Related descriptions should be generally comprehensive and complete, to ensure that the SOP is reproducible by third parties. If necessary, appropriate metadata should be made available, and criteria to evaluate the SOP quality should be given. At the point of data transfer, the generated data are made accessible with the assumption that sensitive data may only be accessible to a restricted circle. Additionally, a persistent identifier is needed that is relevant for subsequent publication. To improve data quality, the persistent identifier should be linked to corresponding SOPs and metadata, e.g., in a standardized format, which could be provided and defined by the MIT. Further developments are necessary to assess the completeness and quality of the data. With regard to ensuring interoperability, SOPs and metadata need to be in a machine-readable format to enable interchangeability across various systems and generate findable metadata (e.g., in a database).

It will be a future challenge to further optimize curation boundaries and develop repositories for data sharing and units for (data) management in line with tasks such as review of requests or corrective actions in the international and multidisciplinary field of nanosafety research.

## Figures and Tables

**Table 1:** Acceptance criteria to be fulfilled within the modules’ biological model and endpoint read out information.

Acceptance Criteria	Brief Description
Source cells	Microscopy observation of cell morphology and viability during cultivation: adherent cell growth and cuboidal cell morphology.
Biological test system	Healthy culture should contain at least 80% viable cells and exhibit a confluence of >70%. Microscopy check of cell morphology and confluence prior to cell cultivation, ENM treatment, and performance of the WST-1 assay.
Viability assay	Corrected absorption of controls representing viable cells between 0.5 and 2, standard deviation of 4 replicates < 0.3.Corrected absorption of cytotoxicity controls should be lower than the viability controls.

**Table 2:** Results of the nested (hierarchical) ANOVA showing type III sum of squares as an estimate of the variation caused by the different factors and their interactions (sources, interactions of two or more factors indicated by *) in comparison to the variation caused by the respective error terms. The degree of freedom (df) values are used to calculate the mean squares of the respective sources, and their ratio provides (hypothesis/error) the F-value.

Source		Type III Sum of Squares	df	Mean Square	F	Sig.
Intercept	Hypothesis	964.142	1	964.142	12.790	0.067
	Error	155.074	2.057	75.382		
Concentration	Hypothesis	286.274	9	31.808	11.748	0.000
	Error	45.014	16.625	2.708		
Replicate	Hypothesis	3.932	2	1.966	2.508	0.181
	Error	3.700	4.720	0.784		
Partner	Hypothesis	148.599	2	74.299	8.988	0.017
	Error	47.589	5.757	8.267		
Experimenter (partner)	Hypothesis	17.565	3	5.855	5.744	0.023
	Error	7.871	7.722	1019		
Concentration * partner	Hypothesis	50.524	18	2.807	6.956	0.000
	Error	7.449	18.459	0.404		
Concentration * experimenter (partner)	Hypothesis	2.879	18	0.160	0.617	0.862
	Error	9.331	36	0.259		
Experimenter * replicate (partner)	Hypothesis	10.674	27	0.395	1.575	0.078
	Error	13.555	54	0.251		
Concentration * partner * replicate	Hypothesis	5.250	6	0.875	3.486	0.006
	Error	13.555	54	0.251		
Concentration * experimenter * replicate (partner)	Hypothesis	9.331	36	0.259	1.033	0.450
	Error	13.555	54	0.251		

**Table 3:** Literature data on NM110 or ZnO ENM IC_50_ values.

Cell Type	Assay	ENM	Incubation Time	Source	IC_50_ (µg/cm^2^)
THP-1	WST-1	NM110	24 h	Safar et al. 2019 [38]	2.53
A549	WST-1	NM110	24 h	Ding et al. 2020 [39]	3.3
A549	WST-1	ZnO (17 nm)	24 h	Remzova et al. 2019 [40]	9.6
A549	WST-1	NM110	24 h	Thongkam et al. 2017 [23]	44

## Data Availability

See Supplementary Materials.

## Supplementary Materials

The following Supporting Information can be downloaded at: https://www.mdpi.com/article/10.3390/nano12071053/s1, Table S1: Minimal information table (MIT); SOP S1: Culturing A549 cells; SOP S2: Sample preparation; SOP S3: Sonicator calibration; SOP S4: Cellular viability—WST-1 assay in A549 cells; Method S1: Background information for the sedimentation analysis; Figure S1: Viability of A549 cells in NM101 (TiO_2_). References [42,43,44,45,46,47,48] are cited in the supplementary materials
