# Supplementary material for: How Structured Metadata Acquisition Contributes to the Reproducibility of Nanosafety Studies: Evaluation by a Round-Robin Test
Source: Nanomaterials (Basel). 2022 Mar 24;12(7):1053. doi: 10.3390/nano12071053 (PMC9000531; doi:10.3390/nano12071053)
Supplement: Supplementary file 1 [file nanomaterials-12-01053-s001.zip › nanomaterials-1627844-SI.pdf]

Supplementary data

How Structured Meta Data Acquisition Contributes to Reproducibility of Nanosafety Studies: Evaluation by a Round Robin Test

Linda Elberskirch [1], Adriana Sofranko [2], Julia Liebing [3], Norbert Riefler [4], Kunigunde Binder [5], Christian Bonatto Minella [5], Matthias Razum [5], Lutz Mädler [4], Klaus Unfried [2], Roel P. F. Schins [2], Annette Kraegeloh [1,*,†] and Christoph van Thriel [3,*,†]

[1] INM—Leibniz Institute for New Materials, Campus D2 2, 66123 Saarbrücken, Germany; linda.elberskirch@leibniz-inm.de
[2] IUF—Leibniz Research Institute for Environmental Medicine, Auf'm Hennekamp 50, 40225 Düsseldorf, Germany; adriana.sofranko@iuf-duesseldorf.de (A.S.); klaus.unfried@iuf-duesseldorf.de (K.U.); roel.schins@iuf-duesseldorf.de (R.P.F.S.)
[3] IfADo—Leibniz Research Centre for Working Environment and Human Factors, Ardeystraße 67, 44139 Dortmund, Germany; liebing@ifado.de
[4] IWT—Leibniz-Institut für Werkstofforientierte Technologien, Badgasteiner Str. 3, 28359 Bremen, Germany; riefler@iwt.uni-bremen.de (N.R.); lmaedler@iwt.uni-bremen.de (L.M.)
[5] FIZ Karlsruhe—Leibniz Institute for Information Infrastructure, Hermann-von-Helmholtz-Platz 1, 76133 Eggenstein-Leopoldshafen, Germany; kunigunde.binder@fiz-karlsruhe.de (K.B.); christian.bonatto-minella@fiz-karlsruhe.de (C.B.M.); matthias.razum@fiz-karlsruhe.de (M.R.)
* Correspondence: annette.kraegeloh@leibniz-inm.de (A.K.); thriel@ifado.de (C.v.T.)
† These authors contributed equally to this work.

Content

Name	Page
**Supplementary SOPs**	1–22
• SOP S1: Culturing A549 cells	1–7
• SOP S2: Sample preparation	8–11
• SOP S3: Sonicator calibration	12–17
• SOP S4: Cellular viability - WST-1 assay in A549 cells	18–22
**Method S1:** Background information to the sedimentation analysis	23
**Figure S1:** Viability of A549 cells to NM101 (TiO_2)	24
**Table S1:** Minimal Information Table (MIT)	25–49

Supplementary SOPs

SOP S1: Culturing A549 cells

NanoS-QM: Standard operating procedure (SOP)
SOP-RRT001-1.0: Culturing A549 cells

SOP information

**Procedure**	Culturing A549 cells
**Scope**	NanoS-QM round robin tests
**Version**	1.0
**Version date**	xx
**SOP ID**	SOP-RRT001-1.0 (Document type: SOP, RRT: round robin tests, SOP Nr. 001, Version 1.0)
**Author**	xx
**Reviewed by**	xx
**Approved by**	xx

SOP history

Version	Approval date	Change description	Changed by
1.0	xx		

1 Introduction

2 Scientific issue

The A549 cell line (DSMZ: A-549, ACC 107) was established in 1972 by D.J. Giard, et al. (Giard et al., 1973). The cells originate from an explant culture of lung carcinomatous tissue from a 58-year-old Caucasian man. This adenocarcinoma human alveolar basal epithelial cell line has been used as a Type II pulmonary epithelial cell model e.g. for drug metabolism (Foster et al., 1998 and others). A549 cells growing adherently as monolayer.

3 SOP purpose

Within the NanoS-QM project, round robin tests with the A549 cell line are carried out. This requires the development of a procedure that allows a standardized cultivation of the A549 cells.

4 Principle of the method

A549 cells are grown in T75 cell culture flasks at 37 °C with 5-10 % CO_2 (Depending on the used cell culture medium). At about 70-90% confluency cells are detached from the flask by Trypsin/EDTA treatment, pelleted and split from 1:5 to 1:10 every 4-7 days in 12 ml complete cell culture medium per T75 flask.

5 Materials

6 Biological characterisation: Cell culture

Information based on the DSMZ Cell Culture Data.
(https://www.dsmz.de/collection/catalogue/details/culture/ACC-107)

Parameter	Information	v/c
Cell line origin / source	DSMZ	c
Provider name	A-549	c
Provider Catalog ID	ACC 107	c
Organism / Species	Human (Homo sapiens)	c
Organ	Lung	c
Tissue	Lung carcinoma	c
Cell type	epithelial	c
Donor sex	man	c
Donor age	58	c
Donor ethnicity	Caucasian	c
Disease	Lung tumor	c
Growth properties	adherent	c
Recommended culture conditions	at 37 °C with 5% CO2	c
Used culture conditions	at 37 °C with 9 % CO_2 (Note: Used DEMEM contains 3,7 g / L sodium bicarbonate. It requires a 8-10 % CO_2 to maintain its physiological pH.)	v
Biosafety level	1	c
Permissions and restrictions	A	c
Reference source	DOI: 10.1093/jnci/51.5.1417; DOI: 10.1002/ijc.2910170110	c

7 Equipment

Parameter	Information	v/c
Cell culture flask	T-75 (Greiner 658 175)<br>T-25 (Greiner 690 175)	v
Centrifuge tubes	TC	v
Sterile disposable pipettes	TC	v
Pipette tips, filter tips	sterile	v
Incubator	Eppendorf Galaxy 48R	v
Centrifuge	Eppendorf 5810R, Rotor A4-62<br>Conditions: 3 min. at 200 x g, brake 9	v
Biological safety workbench	Thermo MaxiSafe 2030i	v
Cell counter	Innovatis Casy TT, cups with 10 ml Cayston	v
Pipettes	Eppendorf	v

8 Reagents

Parameter	Information	v/c
Trypsin/ EDTA	0,05% Trypsin/ 0,02% EDTA, without Ca^{2+}/ Mg^{2+} in PBS<br>PAN P10-23100	v
Buffer	DPBS without Ca^{2+}/ Mg^{2+} (Gibco 141900)	v

9 Cell culture medium

Parameter	Information	v/c
Medium	DMEM (Gibco 31885) + 10 vol% FBS (PAN Biotech)	v

10 Freezing medium

Parameter	Information	v/c
Freezing medium	70% medium, 20% FBS, 10% DMSO	v

11 Procedure

12 Used culture conditions

Parameter	Information	v/c
Cell culture method / Incubation	at 37 °C with 9 % CO_2	v
Cell culturing material	see “Equipment”	v
Medium	90% Dulbecco's MEM (DMEM) + 10% FBS	v
Media preparation	- Completely thawing the FBS<br>- Add FBS to the required percentage to the DMEM bottle (e.g. 500 mL DMEM + 50 mL FBS)<br>- Mix the bottle of media carefully<br>- If needed: Aliquot the media in 50 mL tubes<br>- Label each bottle with the date, media name, percentage of FBS and your name<br>- Storage refrigerated at 2 °C to 8 °C	v
Supplements	-	v
Cell growth / Doubling time	ca. 40 hours	v
Harvest	at 70-80% confluency	v
Cell seeding details / Subculture	seed out at ca. 1-2 x 10^6 cells/75 cm^2; split confluent cultures 1:5 to 1:10 every 4-7 days using trypsin/EDTA<br><br>As a general guide, from a confluent flask of cells:<br>1:2 split should be 70-80% confluent and ready for an experiment in 1 to 2 days.<br>1:5 split should be 70-80% confluent and ready for an experiment in 2 to 4 days.<br>1:10 split should be 70-80% confluent and ready for sub-culturing or plating in 4 to 6 days.	v
Storage	frozen with 70% medium, 20% FBS, 10% DMSO	v
Passage number	3-30 (Passage numbers should be recorded on the flask)	v
Morphology	Epithelial cells, growing adherently as monolayer<br>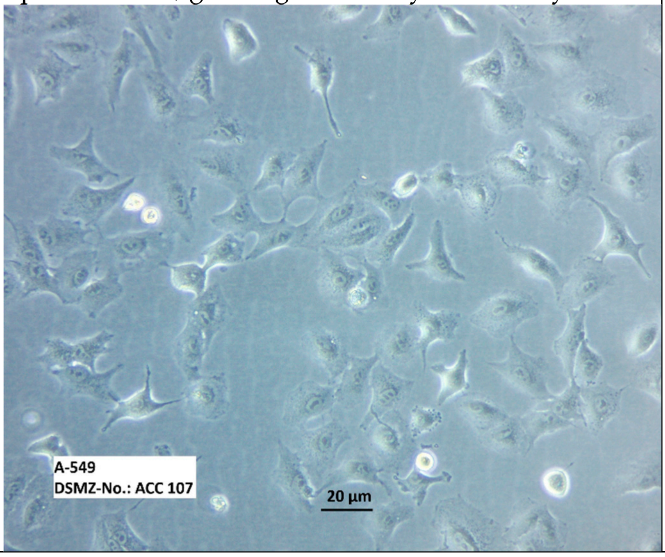	c

	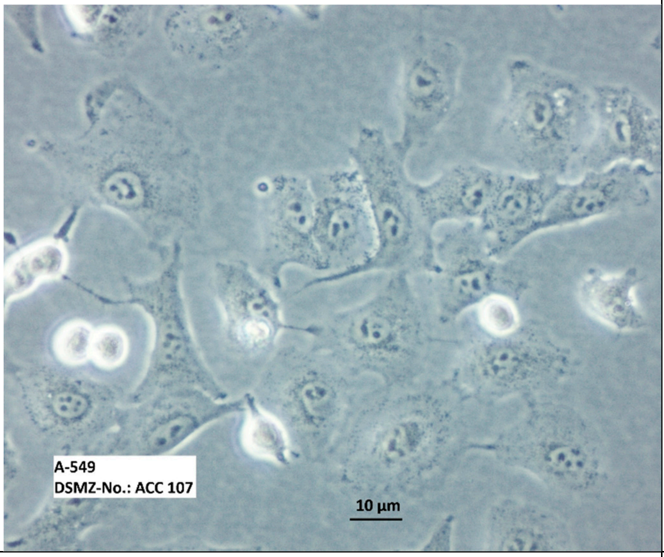	
Differentiation	-	c
Mycoplasma testing (DSMZ)	Negative in DAPI, microbiological culture, RNA hybridization, PCR assays	c

13 Cell thawing

Prearrangements:

- Pre-warm complete cell culture medium at 37°C

Remove cryovial from the liquid nitrogen storage. Thaw cells in a 37°C-water bath until only a small frozen piece is visible. Decontaminate by wipe the vial with 70% Ethanol and transfer cryovial to a sterile hood. All following steps are performed under sterile conditions.

- Transfer entire content of the cryovial into 12 ml pre-warmed (37°C) complete cell culture medium.
- Spin cells down for 5 min at 200 x g at room temperature (RT).
- Discard supernatant, resuspend cells in 12 ml pre-warmed complete cell culture medium and transfer cells into one T75 cell culture flask.
- Incubate at standard growth conditions overnight.
- Check confluency the next day. Cells have to be subcultured (see 14 Subculturing) starting from 70% confluency. In less confluent cultures medium is removed and 12 ml of fresh pre-warmed complete cell culture medium per T75 flask are added and are grown until reaching approximately 70% confluency.
- Culture cells as described below. Passage at least three times before usage in any experiment.

14 Subculturing

Routinely A549 cells are cultured in T-75 flasks in 12 ml complete cell culture medium. They are subcultured every 4-7 days at approximately 70-90 % confluency.

- Pre-warm complete cell culture medium and PBS at 37°C and thaw Trypsin-EDTA
- Discard cell culture medium.
- Wash cells with 10 ml of pre-warmed DPBS (add DPBS to the walls of the flask and carefully distribute over cells to not flush away cells).
- Remove DPBS and for detachment incubate cells with 3 ml Trypsin-EDTA in the incubator for 3-5 minutes.
- Tapping and shaking of the cell culture flask releases the cells from the bottom.

- Add 9 ml of complete cell culture medium, rinse the bottom of the cell culture flask 3-5 times and transfer the resulting 10 ml of cell suspension into a 15 ml conical tube.
- Centrifuge cells for 3 min at 200 x g at RT.
- Discard supernatant.
- Resuspend cell pellet in 1-5 ml of complete cell culture medium by 3-5 gentle pipetting steps. Avoid bubble formation and assure single cell suspension.
- Count cells as described in 15 Cell counting or split ratio 1:5 to 1:10
- Take $5x10^5$ or split ratio cells directly from the cell suspension (volume calculated as described in 4.4 Cell counting). Add up to 12 ml with complete cell culture medium and transfer into one T75 flask.

15 Cell counting

Note: Cell counting can be performed according to each laboratory regular practice. The CASY® TT counting system is described in brief below.

1. Preparing samples
    - Harvest cells as described above (see 14 Subculturing)
    - Fill a CASY cup with 10ml filtered CASYton.
    - Add an aliquot of the cell suspension to be examined (normally between 10µl and 200 µl).
    - Secure the lid and mix the sample by tilting the CASY cup three times, carefully avoiding the formation of bubbles or foam.
2. Measuring the sample
    - Rotate the recess in the sampling platform until it points forward and remove the CASY cup from below the measuring capillary.
    - Place the sample to be measured below the measuring capillary and ensure that the external platinum electrode is immersed in the sample.
    - Rotate the sampling platform somewhat to the side again.
    - Place the protection shield around the sample platform, including the capillary and the external electrode, by pushing it completely to the wall of the sample chamber.
    - Press Measurement select Template and press Measure to perform a measurement.
    - After a short time, the size distribution and the numerical results for your sample appear in the display.

Example template for CASY® TT

Templates represent the measurement method. This means they contain all parameters a CASY uses to analyze a specific sample. Consequently, they are sample specific. Used Template for A549 cells:

Parameter	Information	v/c
Capillary	150 µm	v
Size range	50 µm	v
left-hand normalization cursors	7,13 µm	v
right-hand normalization cursors	30,13 µm	v
left-hand evaluation cursors	11,38 µm	v
right-hand evaluation cursors	30,13 µm	v
Volume	400 µL	v
Cycles	3	v
Dilution	5 * 10^2	v

Mode: Viability or Free Ranges	Viability (will provide a pair of cursors, one to define the dead and one to define the viable cells)	v

16 Cell freezing

To prepare a liquid nitrogen stock A549 cells are expanded until passage 4 after thawing. Grow A549 cells in T75 flasks until subconfluency (~70% confluency) and harvest and freeze them as follows:
Prearrangements:

- Prepare freezing medium and cool down on ice at least one hour before cell harvesting.
- Make sure to have a freezing container on hand.

Cell harvesting:

- Detach cells from the flasks using 2 ml Trypsin-EDTA per flask (see 14 Subculturing).
- Count cells as described in 4.4 "Cell counting" and calculate the number of cryovials needed considering that each vial has to contain 1x106 cells.
- Label each cryovial with cell name, passage number, date and cell number.
- Adjust cell concentration to 1x106 cells/ml in freezing medium. If the initially calculated concentration is higher the suspension is diluted with an appropriate amount of freezing medium. If the initially calculated concentration is lower (e.g. 5x105 cells/ml) cells have to be centrifuged again and resuspended in the correctly calculated volume of the freezing medium.
- Mix well by gently inverting the tube. A homogenous distribution of DMSO as well as cells is important to assure proper freezing and cell viability after thawing.
- Distribute 1 ml of the cell suspension in freezing medium into each cryovial.
- Place cryovials in a freezing container and place this into a freezer (-70°C to -80°C) for 24 h. This leads to a freezing rate of approximately 1°C/min.
- After 24 h place the frozen cryovials into liquid nitrogen for long term storage.

17 Quality criteria

- Calculation of doubling time by cell counts during each subculturing procedure and monitored over time.
- Testing of cell viability after trypsinization. A healthy culture should contain at least 80 % viable cells.

18 Risk assessment

Before starting any experiment A549 cells have to be passaged at least three times after thawing. Furthermore, a maximum of 20 passages (after thawing) must not be exceeded.

19 Cautions

- All procedures have to be carried out under sterile conditions in a laminar flow cabinet (biological hazard standard).
- Only sterile equipment must be used in cell handling. Operators should wear laboratory coat and gloves (according to laboratory internal standards).
- During handling of cryovials in liquid nitrogen, a full-face mask and appropriate gloves must be worn.
- Discard all materials used to handle cells (including remaining cells themselves) according to the appropriate procedure for special biological waste (i.e. by autoclaving)

20 Abbreviation

DSMZ	Deutsche Sammlung von Mikroorganismen und Zellkulturen (engl. German Collection of Microorganisms and Cell Cultures)
DMSO	Dimethyl sulfoxide
FBS	Fetal bovine serum
g	Constant gravitation
PBS	Phosphate buffered saline
v/c	Variable or constant parameter
RT	room temperature

21 Documentation

Please document the following information within the labor protocol for each step and day.

22 Basic information

Parameter	Information
Experimenter	
Date	
Time range	

23 Quality acceptance criteria

Parameter	Information
Doubling time	
Cell viability	

24 Related documents

Document ID	Document title
MIT-RRT001-1.0	Minimal information table: Biological characterisation

25 References

DSMZ: https://www.dsmz.de/collection/catalogue/details/culture/ACC-107

https://www.nanopartikel.info/files/methodik/VIGO/cell_culture_A549.pdf

Foster KA, Oster CG, Mayer MM, Avery ML, Audus KL; 1998: Characterization of the A549 cell line as a type II pulmonary epithelial cell model for drug metabolism. Exp. Cell Res. 243(2): 359-366

Giard DJ, Aaronson SA, Todaro GJ, Arnstein P, Kersey JH, Dosik H, Parks WP; 1973: In vitro cultivation of human tumors: establishment of cell lines derived from a series of solid tumors. J. Natl. Cancer Inst. 51(5): 1417-1423

https://www.ols-bio.de/wp-content/uploads/2020/02/OLS_-CASY_TTT-OperatorsGuide_2018-8.pdf

NanoS-QM: Standard operating procedure (SOP)

SOP-RRT002-1.0: Preparation of nanoparticle dispersions from powdered material using ultrasonic disruption

SOP information

**Procedure**	Preparation of nanoparticle dispersions from powdered material using ultrasonic disruption
**Scope**	NanoS-QM round robin tests
**Version**	1.0<br>Based on: Jensen K.A., The ENPRA dispersion protocol for NANoREG, National Research Centre for the Working Environment, Copenhagen, Denmark (2014)
**Version date**	xx
**SOP ID**	SOP-RRT002-1.0 (Document type: SOP, RRT: round robin tests, SOP Nr. 002, Version 1.0)
**Author**	xx
**Reviewed by**	xx
**Approved by**	xx

SOP history

Version	Approval date	Change description	Changed by
1.0	xx		

26 Scope

This Standard Operating Procedure (SOP) describes the relevant guidelines for the preparation of nanomaterial (NM) dispersions in liquid media by the application of sonication as part of the round robin for examination of quality criteria in work package 3.1 of the NanoS-QM project.

2 Basics

This protocol describes the relevant guidelines for the preparation of engineered NM dispersions in liquid media by the application of ultrasonic disruption, a process referred here as sonication. The engineered NMs are obtained here in dry powder form. Ultrasonic waves are generated liquid media either through direct sonication by inserting the sonicator probe one third into the dispersion or through indirect sonication by inserting the sonicator probe into a bath filled with water surrounding the sample with the dispersion. Indirect sonication requires a higher effective energy output into the suspension as the ultrasonic waves must cross the bath water and then pass through the wall of the sample container before reaching the suspension. These physical barriers can be reduced by direct sonication where the sonicator probe is inserted directly into the suspension.

3 Materials and Instruments

a. Materials

Sterile pipette tips

Polystyrene Conical Centrifuge Tube 50 ml (BD Falcon #352070)
Disperser 5 ml (Band PD-Tips #702390) und 0.5 ml (Brand PD-Tips #702384)
Rotisolv HPLC gradient water (Roth #A511.2) or Nanopure-filtered water or MilliQ-filtered water or similar; resistivity 18.2 MΩ cm)
600 mL Borosilicate glass beaker, tall form (height 150 mm and 80 mm in diameter), with spout. (similar to VWR catalogue number 213-1174)
Nanomaterials NM101 and NM110
FCS / FBS

b. Instruments
Single channel pipette
Vortexer
Laboratory balance
Probe sonicator
Digital thermometer with measurement accuracy better than ±0.1°C
Styrofoam box

4. Preparations

- All materials are supplied as dry powders
- All vials should be stored at room temperature, in darkness and dry
- BE FAST, once the material vial is open!

Nanomaterials used in this SOP

Material	NM code	Label name	label second line	CAS-Number	Source
TiO_2	NM-101	Titanium Dioxide, thermal photocat 7	rutile-anatase, thermal, 7 nm	13463-67-7	Hombikat UV100
ZnO uncoated	NM-110	Zinc Oxide	uncoated 100 nm	1314-13-2, *EINECS* 215-222-5	BASF Z-Cote

- Disperse the nanomaterial (NM) in HPLC water supplemented with 2% fetal calf serum (FCS)
- Water with serum (=dispersion media) can be stored frozen at -20 °C for long term storage
- Weigh app. 15.36 mg particles corresponding to 6 ml dispersion media in a 50 mL Falcon Tube. This will give a **concentration of 2.56 mg/ml**
    - Turn on a ventilation hood and wait for 20 second until the air flow safe. Then turn on the balance for weighing the particles.
    - Weigh the vial for the stock suspension with cap
    - Remove cap from suspension vial
    - Remove cap from material vial
    - Transfer material to stock suspension vial (at least 15.36 mg NM for 6 ml dispersion)
    - Close the material vial
    - Close the stock suspension vial
    - Weigh the stock suspension vial and calculate mass difference
    - Add calculated amount of dispersion media to get a concentration of 2.56 mg/ml
    - Sonicate particles as follows:

- Sonicate particles with a Sonifier equipped with a standard disruptor horn.
- Cool the samples continuously in an ice-water bath to prevent heating of the samples (see Scheme S2).

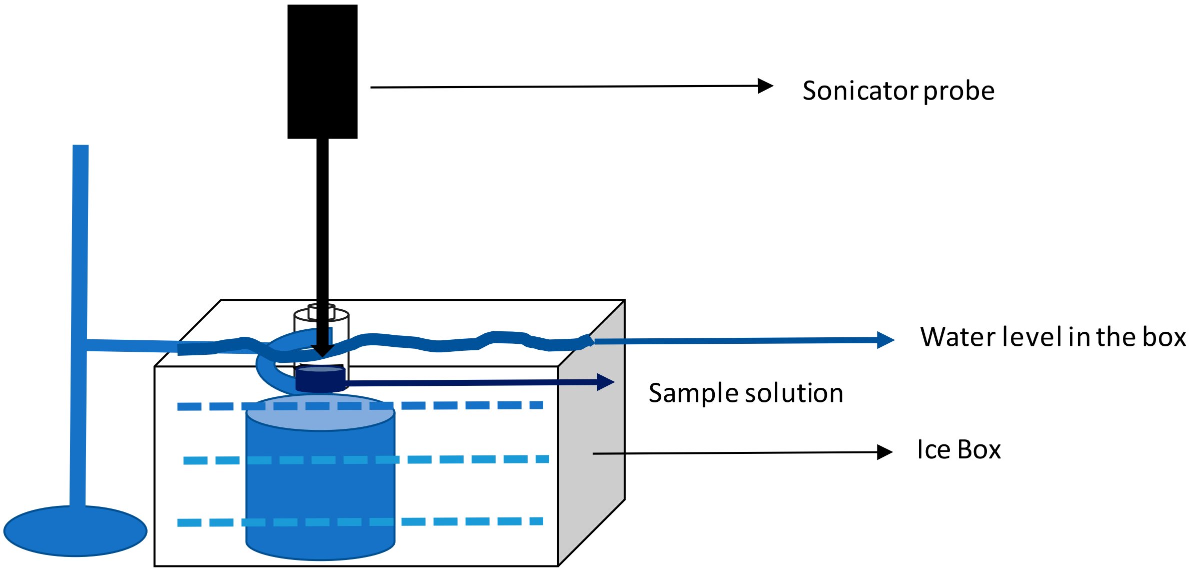

Sonication set up for placement of sonication vial in ice-water bath (modified from Jensen 2014, NANoREG).

- Fill a 250 ml glass beaker with ice and place it upside-down in a styrofoam box
- Add 85-90 vol% ice into the insulation box
- Add. 10-15 vol% cold water into the insulation box
- Place the sample vial on top of an upside-down glassbeaker and fill the Styrofoam box with the ice-water to keep the dispersion cooled.
- Fix the vial using a clip or burette holder
- Insert the sonicator probe one third into the dispersion.
- Keep attention that the sample vial does not touch the wass of the scintillation vial.
- Sonicate for 16 min at the settings identified of your specific probesonifier using the *NANoREG SOP for for probe-sonicator calibration of delivered acoustic power and de-agglomeration efficiency for in vitro and in vivo toxicological testing*
- Clean the sonication probe by sonication for 5 minutes with the probe fully immersed in a 50:50 water-ethanol mixture followed by rinsing in ethanol using a dispenser and airdry the probe. Other in-house cleaning methods may also apply.
- Following the sonication step, transfer all samples immediately to ice
- Utilize test suspensions within 60 minutes

6. Safety precautions

Follow the safety information and regulations of the working laboratory.

7. Waste disposal

Follow the disposal advice from materials providers, if available.

8. References

Jensen K.A., The ENPRA dispersion protocol for NANoREG, National Research Centre for the Working Environment, Copenhagen, Denmark (2014)

SOP S3: Sonicator calibration;

NanoS-QM: Standard operating procedure (SOP)

SOP-RRT003-1.0: Probe sonicator calibration

SOP information

**Procedure**	Probe sonicator calibration
**Scope**	NanoS-QM round robin tests
**Version**	1.0<br>Based on: NANoREG D4.12 SOP Probe Sonicator Calibration for ecotoxicological testing
**Version date**	xx
**SOP ID**	SOP-RRT003-1.0 (Document type: SOP, RRT: round robin tests, SOP Nr. 003, Version 1.0)
**Author**	xx
**Reviewed by**	xx
**Approved by**	xx

SOP history

Version	Approval date	Change description	Changed by
1.0	xx		

1. Scope

This Standard Operating Procedure (SOP) describes the relevant guidelines for the probe sonicator calibration in liquid media by the measurement of temperature increase as part of the round robin for examination of quality criteria in work package 3.1 of the NanoS-QM project.

2. Basics

This protocol describes the relevant guidelines for the probe sonicator calibration in liquid media for engineered NM dispersions in liquid media by the application of ultrasonic disruption, a process referred here as sonication. The engineered NMs are obtained here in dry powder form. All preparations and subsequent characterization procedures should be as harmonized as possible to obtain comparable aqueous NM dispersions for the rate of agglomeration and sedimentation. The ultrasonic energies can differ by different brands of sonifiers, water qualities, sample volume, concentration and working temperatures, which may have an effect on the results.

3. Materials and Instruments

a. Materials

Sterile pipette tips
Polystyrene Conical Centrifuge Tube 50 ml (BD Falcon #352070)
Disperser 5 ml (Band PD-Tips #702390) und 0.5 ml (Brand PD-Tips #702384)
Rotisolv HPLC gradient water (Roth #A511.2) or Nanopure-filtered water or MilliQ-filtered water or similar; resistivity 18.2 MΩ cm)
600 mL Borosilicate glass beaker, tall form (height 150 mm and 80 mm in diameter), with spout. (similar to VWR catalogue number 213-1174)

b. Instruments

Single channel pipette

Vortexer

Laboratory balance with maximum weight boundary greater than 700 g and a weighing accuracy of ±0.1 g or better

Probe sonicator

Digital thermometer with measurement accuracy better than ±0.1°C

4. Specific Considerations

a. Temperature

During sonication, the dispersion can be heated over time by the extreme local heating cycles of the sonifier. To minimize temperature increase, the sample vial should be immersed in a container or box containing ice water. The material of the container can be as follows: aluminum, stainless steel, glass or plastic.

b. Sonication time

The total amount of energy (E) delivered to a suspension depends on the applied power (P) and the total amount of time (t): $E = P \times t$.

c. Sample volume and concentration

Samples with different volumes and particle concentrations can respond differently to the same amount of delivered energy. The effect of sample volume at equal particle concentrations is measured as energy density (W·s/mL). This magnitude expresses the amount of delivered energy per unit of suspension volume. At equal power and particle concentration, higher energy densities in lower suspension volumes will result in higher disruptive effect.

d. Sonicator probe and tip immersion

The amount of acoustic energy transferred to the suspension is dependent on the shape and diameter of the probe and its immersion depth into the suspension. Sonicator probe immersion depths between (2 to 5) cm are recommended and should be placed no closer than about 1 cm from the bottom of the sample container, and contact between the probe and the container walls should be avoided.

e. Media

The ultrasonic energy delivered to a suspension is dependent by the medium's viscosity, density and chemical composition.

5. Calibration of delivered power from calorimetry

Calorimetry is a procedure that allows for the direct measurement of the effective acoustic energy delivered to a sonicated liquid. The method is based on the measurement of the temperature increase in a liquid medium over time according to the following protocol (based on NANoREG D4.12 SOP Probe Sonicator Calibration for ecotoxicological testing):

1. Fill HPLC, nanopure or MilliQ-filtered water (or similar quality with resistivity of 18.2 MΩ cm) into a large glass flask (it is recommended to draw at least 3 L so you have enough for at least 6 runs).
2. Place the large glass flask in the fume hood until the water reaches the fume hood temperature. This will normally take a few hours!
3. Fill a 600 mL glass beaker with 500 mL of the thermally equilibrated water

a. Measure and document the temperature using a digital temperature sensor with an uncertainty of less than 0.1°C.

b. Tare with an empty beaker using a top load balance and determine the mass of the 500 mL water with an uncertainty of less than ±0.1 g.

4. Place the 600 mL beaker in the sonicator chamber and immerse the sonicator probe approximately 2.5 cm below the liquid surface.

a. Stabilize the beaker using a hook/clamp during the sonication and measurement. See example in Scheme S1.

5. Immerse a temperature probe connected to a temperature meter and data logger at a depth of 2.5 cm and 1 cm away from the sonicator probe. See example in Scheme S1.

a. The temperature should be measured with an uncertainty smaller than 0.1°C.

b. Stabilize the temperature probe using a clamp so it will stay at the same position and distance from the tip of the sonicator probe.

6. Select the lowest sonicator output setting with effect (e.g. "amplitude" or "% of amplitude"; usually set by a dial in the sonicator power module). Operate it in continuous mode and record the water temperature increase for the initial 6.5 minutes with a time-resolution of no more than 30 seconds.

Perform the experiment for the start condition twice before continuing to point 7 (use a new 600 mL for each experiment!)

7. Increase the amplitude to ca. 20% of the maximum and repeat the test described under point 6 (use a new 600 mL beaker!). Record the water temperature increase for the initial 6.5 minutes with a time-resolution of no more than 30 seconds.

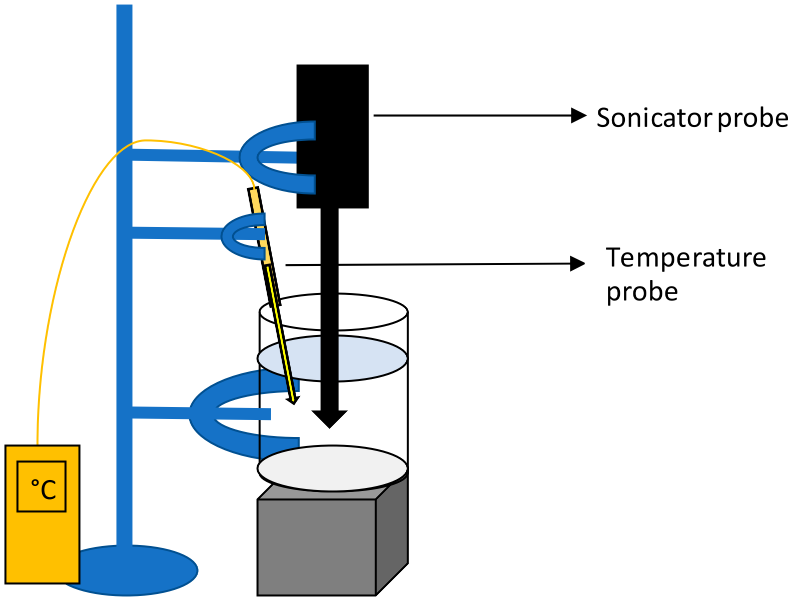

Scheme S1. Setup for the measurement of probe sonication calorimetric curves

8. Fill in an excel table the requested sonicator data, experimental data and conditions, the recorded temperature and time data for the lowest and ca. 20% maximum amplitude settings. Using the recorded values, create a temperature vs. time curve and obtain the best linear fit for the curve using least squares regression to calculate the delivered acoustic power (Pac) using the calorimetric approach:

a. Perform quality check of the measured temperature profiles from the automatically generated temperature-time diagrams. Check that the time-temperature curve appears to have the same slope from the first measurement and onwards. The linear fit should have a R^2-value > 0.990 between the measured temperature in °C as function of time in seconds (both repeated data

sets) using least squares regression. (Data are illustrated in Scheme S2). If these criteria are not fulfilled, a new data-set may be required.

b. At a given device output power setting, the temperature increase in the liquid is recorded over time and the effective delivered power can be calculated using the following equation:

$$P_{ac}(Watt) = \frac{\Delta T}{\Delta t} MC_p$$

where P is the delivered acoustic power (W), ΔT/Δt is the slope of the regression curve, T and t are temperature (K) and time (s), respectively, Cp is the specific heat of the liquid (4.18 J/g*K for water) and M is the mass of liquid (g).

The P_{ac} values for each run are given in cell D53, E53, and F53 in the Excel template.

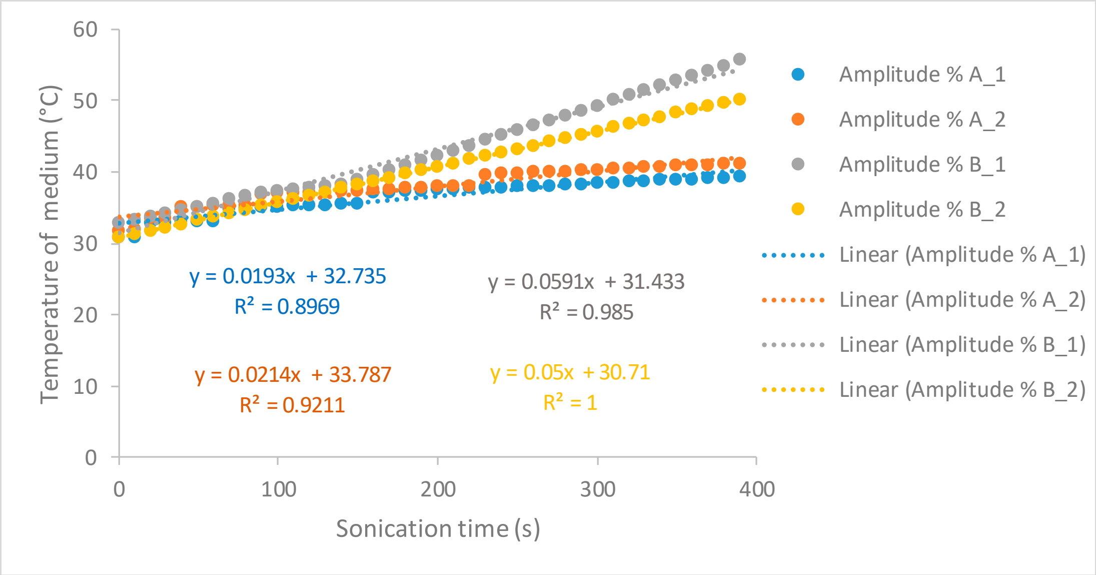

Scheme S2. Temperature increase as function of sonication time using a 20kHz Branson S-450D with a 13 mm disruptor horn at different sonicator probe amplitude (10 and 20%) settings.

9. Determine the third setting for estimation of the correct amplitude settings like this:

a. If the P_{ac} for the lowest and 20% amplitude setting are both lower than the required 7.35±0.05 Watt, project the required amplitude setting (assume linear relationship) to reach the requested 7.35±0.05 Watt. Repeat the test described under point 6 with the amplitude setting obtained by extrapolation twice (use a new 600 mL for each experiment!).

b. If the P_{ac} for the minimum and 20% amplitude setting is lower and higher than 7.35±0.05 Watt, respectively, interpolate (assume linear relationship) the amplitude setting to achieve the requested acoustic power of the probe. Repeat the test described under point 6 (use a new 600 mL beaker!) with the amplitude setting obtained by interpolation extrapolation twice (use a new 600 mL for each experiment!).

c. If the P_{ac} for the lowest amplitude setting is higher than 7.35±0.05 Watt, then calculate the sonication time (t in s) to deliver a total energy of 7056±103 J using the equation: t(s) = 7056 J/P_{ac}(W). Use the P_{ac} value which was determined for your lowest amplitude setting for the calculation.

Chart S1. Guidelines for optimization starting points.

Energy Density (W·s/mL)	Sample Volume (mL)	Power (W)	Time (s)
Below 100	10	50	Below 20
100 to 500	10	50	20 to 100
Over 500	10	50	Over 100

10. Enter the requested sonicator data, experimental data and conditions, the recorded temperature and time data for the third amplitude setting tested in point 9 in the second sheet of the excel file to automatically plot and calculate the delivered acoustic power (P_{ac}) for this last setting. Again, P_{ac} for each run are given in cell D53 and E53 in the Excel template.

11. Go to the sheet 3rd sheet in the "Calibration of delivered power from calorimetry" Excel file to identify the setting required to deliver Pac = 7.35±0.05. Watt. The "Amplitude Estimation" sheet contains the calculated P_{ac} and a column for your amplitude settings and a graph with a regression curve for the amplitude versus the P_{ac} in your test (see example Scheme S3).

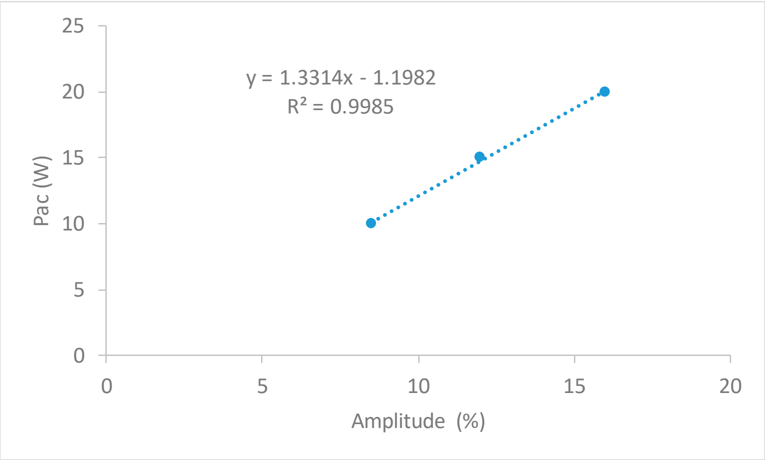

Scheme S3. Probe amplitude setting plotted as function of calculated delivered acoustic sonication power P_{ac} (in this example: percent amplitude for the Branson 450). The linear regression function can be used to calculate the setting required to reach the required delivered acoustic sonication power.

Decision tree for selection of amplitude settings

a. If the sonicator is able to produce a Pac = 7.35±0.05 Watt, use the calculated output setting.
b. If the maximum amplitude setting of the sonicator does not reach P_{ac} = 7.35±0.05 Watt, calculate the sonication time (t in s) to deliver a total 7056±103 J using the equation t(s) = 7056 J/P_{ac}(W). Use the P_{ac} value which was determined for your highest amplitude setting for the calculation. 7056±103 J corresponds to the total delivered acoustic energy in the ENPRA and NANOGENOTOX dispersion protocols over a 16 min period.

6. Safety precautions

Follow the safety information and regulations of the working laboratory.

7. Waste disposal

Follow the disposal advice from materials providers, if available.

8. References

Booth A. and Jensen K.A., SOP for probe sonicator calibration of delivered acoustic power and deagglomeration efficiency for ecotoxicological testing (2015). http://creativecommons.org/licenses/by-nc-sa/4.0/

NanoS-QM: Standard operating procedure (SOP)

SOP-RRT004-1.0: Cellular viability - WST-1 assay in A549 cells

SOP information

**Procedure**	Cellular viability - WST-1 assay
**Scope**	NanoS-QM round robin tests
**Version**	1.0
**Version date**	xx
**SOP ID**	SOP-RRT004-1.0 (Document type: SOP, RRT: round robin tests, SOP Nr. 004, Version 1.0)
**Author**	xx
**Reviewed by**	xx
**Approved by**	xx

SOP history

Version	Approval date	Change description	Changed by
1.0	xx		

1. Scope

This Standard Operating Procedure (SOP) describes the analysis of the cellular toxicity of nanomaterials via WST-1 assay as part of the round robin for examination of quality criteria in work package 3.1 of the NanoS-QM project.

2. Basics

The cell proliferation reagent WST-1 is designed to be used for the non-radioactive, spectrophotometric quantification of cell proliferation, growth, viability, and chemosensitivity in cell populations using the 96-well-plate format. The assay is based on the cleavage of tetrazolium salts to formazan by cellular enzymes. An expansion in the number of viable cells results in an increase in the overall activity of mitochondrial dehydrogenases, and the formation of formazan is directly proportional to the number of metabolic active cells in the culture. The aim of this SOP is to assess nanomaterials' cytotoxicity using the WST-1 assay. In order to eliminate possible interferences from nanomaterials with the absorbance readings and/or with the WST-1 substrate, the standard procedure from the reagent provider has been adapted by Vietti et al. 2013 (doi:10.1186/1743-8977-10-52).

3. Materials and Instruments

a. Materials

- Sterile pipette tips
- 96-well microplates, flat bottom, sterile (BD Falcon #353072)
- Culture media DMEM (Gibco, Thermo #31885023)
- Culture media DMEM without phenol red (Gibco, Thermo #11054020)
- WST-1 Stock Solution (2-(4-iodophenyl)-3-(4-nitrophenyl)-5-(2,4-disulphophenyl)-2H-teterazolium, Roche Diagnostics, #11 644 807 001) - stored in 1mL aliquots at -20°C

- Triton X-100, CAS 9002-93-1 (Sigma #T8787)
- Sterile PBS (Sigma #D8537)
- Cell line in culture
- Polystyrene Conical Centrifuge Tube 15 ml (BD Falcon #352095)
- Polystyrene Conical Centrifuge Tube 50 ml (BD Falcon #352070)
- Disperser 5 ml (Band PD-Tips #702390) und 0.5 ml (Brand PD-Tips #702384)
- Rotisolv HPLC gradient water (Roth #A511.2)

b. Instruments

- Microscope
- Hemacytometer
- Multistep pipette
- Single channel pipette
- Incubator (37oC, 5% CO2, humidified atmosphere)
- Centrifuge
- Scanning multiwell spectrophotometer with filters for 450 nm (at least between 420 to 480 nm) and a reference filter for 630nm (at least above 600).
- Biological safety cabinet, class 2
- Vortexer
- Water bath 37°C

4. Experimental procedure

a. Cell culture

- Seed cells in an appropriate cell density in a 96-well plate (Chart S2). For most experimental setups, a cell concentration between 0.1 and 5 x 10^4/well and an incubation time of 24 to 96 hours is appropriate. Cell treatments should be done at 70% confluence

Chart S2. Cultivation conditions for A549 cells

Cell line	Cell density	Cultivation time	Culture medium	Assay medium	WST-1 Incubation
A549	10,000 cells/well	24 h	DMEM (+ phenol red) +10% FCS	DMEM (- phenol red) (- glutamine) +1% L-glutamine +10% FCS	0.5 h

- Culture cells in 96-well plate in 100 µl medium per well for 24 hours
- Run samples as six replicates
- Avoid all marginal wells (Scheme S4, green panels) as cells grow differently
- Use the outer wells of the plate as blank controls (Scheme S4, row A and row H)

	1	2	3	4	5	6	7	8	9	10	11	12
A		0 µg/cm^2	.3125 µg/cm	.625 µg/cm	1.25 µg/cm^2	2.5 µg/cm^2	5 µg/cm^2	10 µg/cm^2	20 µg/cm^2	40 µg/cm^2	80 µg/cm^2	
B		NM110	NM110	NM110	NM110	NM110	NM110	NM110	NM110	NM110	NM110	
C		↓	↓	↓	↓	↓	↓	↓	↓	↓	↓	
D		↓	↓	↓	↓	↓	↓	↓	↓	↓	↓	
E		↓	↓	↓	↓	↓	↓	↓	↓	↓	↓	
F		↓	↓	↓	↓	↓	↓	↓	↓	↓	↓	
G		↓	↓	↓	↓	↓	↓	↓	↓	↓	↓	
H		↓	↓	↓	↓	↓	↓	↓	↓	↓	↓	

without cells — plus 0.5% Triton-X

	1	2	3	4	5	6	7	8	9	10	11	12
A		0 µg/cm^2	.3125 µg/cm	.625 µg/cm	1.25 µg/cm^2	2.5 µg/cm^2	5 µg/cm^2	10 µg/cm^2	20 µg/cm^2	40 µg/cm^2	80 µg/cm^2	
B		NM101	NM101	NM101	NM101	NM101	NM101	NM101	NM101	NM101	NM101	
C		↓	↓	↓	↓	↓	↓	↓	↓	↓	↓	
D		↓	↓	↓	↓	↓	↓	↓	↓	↓	↓	
E		↓	↓	↓	↓	↓	↓	↓	↓	↓	↓	
F		↓	↓	↓	↓	↓	↓	↓	↓	↓	↓	
G		↓	↓	↓	↓	↓	↓	↓	↓	↓	↓	
H		↓	↓	↓	↓	↓	↓	↓	↓	↓	↓	

without cells — plus 0.5% Triton-X

Scheme S4. Plate design for a WST-1 assay

b. Preparations

- All materials are supplied as dry powders
- All vials should be stored at room temperature, in darkness and dry
- BE FAST, once the material vial is open!

Chart S3. Nanomaterials used in this SOP

Material	NM code	Label name	label second line	CAS-Number	Source
TiO_2	NM-101	Titanium Dioxide, thermal photocat 7	rutile-anatase, thermal, 7 nm	13463-67-7	Hombikat UV100
ZnO uncoated	NM-110	Zinc Oxide	uncoated 100 nm	1314-13-2, *EINECS* 215-222-5	BASF Z-Cote

- Disperse the nanomaterial (NM) in HPLC water supplemented with 2% fetal calf serum (FCS) following the steps described in the "SOP-Probenpräparation" using a Sonifier equipped with a standard disruptor horn.
- Utilize test suspensions within 60 minutes

c. Treatment

- Prepare NM test suspensions or positive controls (Chart S4)
- The stock suspension 2.56 mg/ml (particles in water with 2% FCS should be diluted 10 times with full normal cell media
- **Highest tested concentration will thus be 256 µg/ml**

Chart S4. Pipette scheme for different nanomaterial suspensions and positive controls.

NM dilution	in µg/ml	NM dilution	Stock suspension [µl]	Dispersion media (µl)
**80 µg/cm²**	256 µg/ml	1	2000	0
**40 µg/cm²**	128 µg/ml	2	1000 of 1	1000
**20 µg/cm²**	64 µg/ml	3	1000 of 2	1000
**10 µg/cm²**	32 µg/ml	4	1000 of 3	1000
**5 µg/cm²**	16 µg/ml	5	1000 of 4	1000
**2.5 µg/cm²**	8 µg/ml	6	1000 of 5	1000
**1.25 µg/cm²**	4 µg/ml	7	1000 of 6	1000
**0.625 µg/cm²**	2 µg/ml	8	1000 of 7	1000
**0.3125µg/cm²**	1 µg/ml	9	1000 of 8	1000
**0 µg/cm²**	0 µg/ml	10	0	1000

- Prepare Dispersion media without NMs for the NM dilution steps (dilute HPLC water supplemented with 2% FCS 1:10 in full cell culture media, e.g. 9 ml cell culture media + 1 ml HPLC with 2%FCS)
- Prepare ten different treatment concentrations using 2-fold serial dilutions of the materials, with the highest dose of 80 µg/cm²
- Rinse wells with 100 µl/well with normal cell media without phenol red
- Incubate cells with test materials in 100 µl/well in normal cell media without phenol red at 37°C, 5% CO_2 for 24 hours

d. Incubation with WST-1

- After a 24-hour-incubation, expose two of the six replicates to 0.5% Triton-X-100 in serum-free medium without phenol red (Scheme S4, red frames)
- Incubate plates for 15 minutes at 37°C and 5% CO_2
- Add 10 µl of WST-1 solution/well
- Incubate cells at 37°C and 5% CO_2, for incubation times see Chart S2

e. Plate reading

- Shake plate for one minute and measure absorbance of the samples using a measure wavelength at 450 nm and a reference wavelength at 630 nm

f. Data analysis

- The average absorbance of the blanks (WST-1 without cells) is subtracted from sample absorbance at 450 nm and absorbance are corrected by their respective reference
- Corrected absorbance of dead cells is subtracted from live cells corrected absorbance
- Results are expressed relative to medium control
- Three independent experiences using 4 replicates should be reported

g. Test acceptance criteria

Absorbance at 450 nm - absorbance at 630nm of:

- $A_{450\,nm}$- $A_{630\,nm}$ of medium controls (cells incubated in culture media without phenol red) should be between 0.5 and 2, standard deviation should be <0.3.
- Positive controls (cells exposed to Triton-X-100) should be lower than the controls

27 . Safety precautions

Follow the safety information and regulations of the working laboratory and of materials providers. Biosafety level 1 precautions should be followed when handling cells.

28 . Waste disposal

Follow the disposal advice from materials providers, if available. Any material containing cells should be discarded as bio hazardous waste.

29 . References

Booth A. and Jensen K.A., SOP for probe sonicator calibration of delivered acoustic power and deagglomeration efficiency for ecotoxicological testing (2015). http://creativecommons.org/licenses/by-nc-sa/4.0/

Jensen K.A., The ENPRA dispersion protocol for NANoREG, National Research Centre for the Working Environment, Copenhagen, Denmark (2014)

Vietti, G., Ibouraadaten, S., Palmai-Pallag, M. et al. Towards predicting the lung fibrogenic activity of nanomaterials: experimental validation of an in vitro fibroblast proliferation assay. Part Fibre Toxicol 10, 52 (2013). https://doi.org/10.1186/1743-8977-10-52

Method S1: Background information to the sedimentation analysis

To quantitatively compare the measured decline of the absorption of the UV-Vis spectra, the analytical concentration distributions c(z) of the nanoparticles along the vertical axis due to gravity at equilibrium are calculated. The particle distribution is an exponential decay curve depending on the vertical position z, calculated according to Midelet et al. [Midelet 2017]:

$$c(z) = c_0 Bexp(-z/z_0)$$

where z_0 is the characteristic height of the equilibrium gradient:

$$z_0 = 24k_B T/(4\pi g {d_p}^3(\rho - \rho_{liq}))$$

with the Boltzmann constant k_B, temperature T, gravitational constant g, particle diameter d_p and the densities of the particles rho and of the liquid rho_{liq}, respectively. The concentration factor is given by:

$$B = z_{max}/(z_0(1 - exp(z_{max} - z_0)))$$

Using density and viscosity values from [Croughan 1989] and a sedimentation length of z_{max} = 25 mm, i.e. the liquid filling height within a cuvette, concentration distributions at equilibrium shown in Scheme S5 for different TiO_2 particle diameters indicates at z = 25 mm (i.e. the top of the liquid column in a conventional cuvette) for small particles a high particle concentration, while larger particles are going to sediment much more.

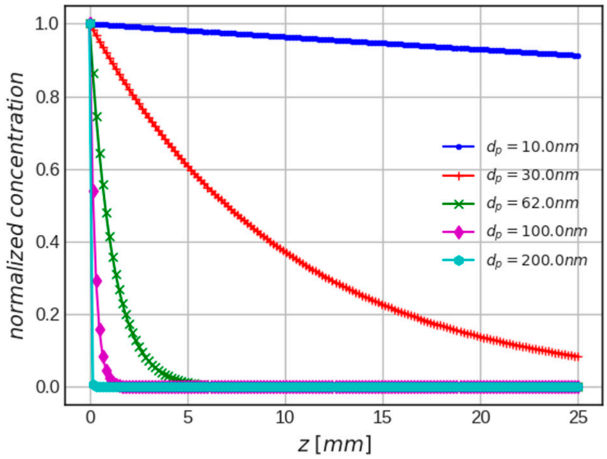

Scheme S5. Concentration drops at equilibrium depending on the nanoparticle diameter. Accordingly, aggregates and agglomerates also exhibit a more pronounced tendency to sediment as compared to separate particles.

These theoretical curves vary only little, within a few percent for different fluids or if the particle density changes from TiO_2 (3.9 g/cm^3) to ZnO (5.6 g/cm^3), therefore the key parameter is the particle diameter. However, these curves represent equilibrium states and their forming - expressed by the equilibrium sedimentation time - require long time spans up to months in case of small particles. Additionally, it is not clear to which extent the particles undergo agglomeration, especially in presence of biological media, which would lead to considerably faster sedimentation times.

Figure S1: Viability of A549 cells to NM101 (TiO_2)

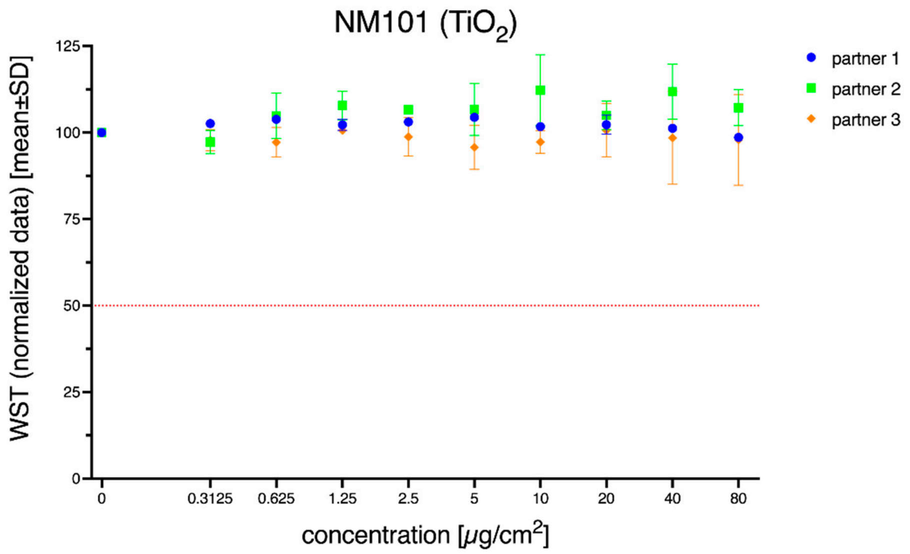

Table S1: Minimal Information Table (MIT)

Source: Elberskirch, L.; Binder, K.; Riefler, N.; Sofranko, A.; Liebing, J.; Minella, C.B.; Mädler, L.; Razum, M.; van Thriel, C.; Unfried, K.; et al. Digital research data: from analysis of existing standards to a scientific foundation for a modular metadata schema in nanosafety. *Part. Fibre Toxicol.* **2022**, *19*, 1–19, doi:10.1186/S12989-021-00442-X/FIGURES/4.

1 General information

Parameter	Parameter description	Partner 1	Partner 2	Partner 3
Experiment name	Short and descriptive title [OECD GD 211]	Preparation of nanoparticle dispersions from powdered material using ultrasonic disruption and Cellular viability - WST-1 assay in A549 cells	NanoS-QM Ringversuch WST-1	Cellular viability - WST-1 assay in A549 cells
Study description	A description of why you did the experiment, what you hoped to see. This should be a 2-3 sentence description that would explain what this experiment is to someone other than you. [MIFlowCyt]	The assay is based on the cleavage of tetrazolium salts to formazan by cellular enzymes. An expansion in the number of viable cells results in an increase in the overall activity of mitochondrial dehydrogenases, and the formation of formazan is directly proportional to the number of metabolic active cells in the culture. The aim of this SOP is to assess nanomaterials' cytotoxicity using the WST-1 assay. In order to eliminate possible interferences from nanomaterials with the absorbance readings and/or with the WST-1 substrate, the standard procedure from the reagent provider has been adapted by Vietti et al. 2013 (doi: 10.1186/1743-8977-10-52).	The analysis of the cellular toxicity of nanomaterials via WST-1 assay as part of the round robin for examination of quality criteria in work package 3.1 of the NanoS-QM project.	Cells were seeded in an appropriate cell density in culture medium in a 96-well plate. Cell treatments could take place at 70% confluence.
Date	Report the date of the first version of the method description (day/month/year). Example: "5 August 2013" [OECD GD 211]	xx	xx	xx
Organizations	Organization(s) performing the experiment. [MIFlowCyt]	xx	xx	xx
Experimenter(s)	Name and email of contact person, Name of further persons involved [ToxTemp]	xx	xx	xx

Time point	Date and time (range) of the experiment [OECD GD 211]	xx	xx	xx
PubMed IDs	A numeric PubMed ID of a publication associated with this experiment or a comma-separated list on multiple numeric PubMed IDs if multiple publications are related to this experiment. [MIFlowCyt]	Results not published yet		
PMC IDs	A PMC publication identifier (i.e., PMCxxxxxxxx) of a publication associated with this experiment, or a comma-separated list of multiple PMC IDs if multiple publications are related to this experiment. [MIFlowCyt]	Results not published yet		
DOI	DOI stands for Digital Object Identifier. It is a unique ID to correspond to an electronic document such as an electronic journal article. Note, not all electronic journal articles have a DOI. However, you may come across this ID on a journal article page, on a publisher's website, in a record in a database such as PubMed, or in a citation in a bibliography. Some citation styles include the DOI at the end of the citation. [Health and science library]	Results not published yet		
Reference(s) to main scientific papers	List of bibliographic references to papers that explain assay development [OECD GD 211]	Vietti, G., Ibouraadaten, S., Palmai-Pallag, M. et al. Towards predicting the lung fibrogenic activity of nanomaterials: experimental validation of an in vitro fibroblast proliferation assay. Part Fibre Toxicol 10, 52 (2013). https://doi.org/10.1186/1743-8977-10-52	A Multilaboratory Toxicological Assessment of a Panel of 10 Engineered Nanomaterials to Human Health—ENPRA Project—The Highlights, Limitations, and Current and Future Challenges; Kermanizadeh et al. 2016; To link to this article: http://dx.doi.org/10.1080/10937404.2015.1126210	
Test type	e.g., in vivo, or in vitro, ex vivo, in chemico, in silico	in vitro		

Status of the method	i) Development status: Indicate if the assay is still under development, and the estimated timeline for completion as far as possible; ii) Known uses: summarise the current and/or past use of the assay by different laboratories; iii) Evaluation study: summarise the main conclusions or refer to individual protocol if available; iv) Validation study: indicate participation in a formal validation study/studies and summarise the conclusions and their outcomes or refer to the individual protocol if available; v) Regulatory use: provide details of any potential regulatory application and of the toxicological hazard endpoint being addressed by the assay [OECD GD 211]		WST-1 assay in principal finished, also with A549 but during the experiment adaption and development of the SOP	SOP was adjusted during the study
Standards followed	Standards followed such as good cell culture practice [OECD GD 211]	none		
Ethical statement	For experiments involving live vertebrates, include a statement of compliance with ethical regulations and identify the committee(s) approving the experiments. [Nature]	none		
Abbreviations and definitions	Used abbrevations and definitions of specific terminology	Standard operating procedure (SOP)<br>WST-1 (2-(4-iodophenyl)-3-(4-nitrophenyl)-5-(2,4-disulphophenyl)-2H-teterazolium,	WST-1 (2-(4-iodophenyl)-3-(4-nitrophenyl)-5-(2,4-disulphophenyl)-2H-teterazolium	DSMZ (Deutsche Sammlung von Mikroorganismen und Zellkulturen (engl. German Collection of Microorganisms and Cell Cultures))<br>DMSO (Dimethyl sulfoxide)<br>FBS (Fetal bovine serum)<br>g (Constant gravitation)<br>PBS (Phosphate buffered saline)<br>RT (room temperature)

2 Biological model information

Parameter	Parameter description	Partner 1	Partner 2	Partner 3
Cell name	Immortalized (naturally or engineered), genetically uniform tissue cells able to reproduce indefinitely in standard culture conditions. [LINCS]		A549	
Provider name	Name of vendor or lab (provider) that supplied the cell line [LINCS]		DSMZ	
Provider Catalog ID	ID or catalogue number or name assigned to the cell line by the vendor or provider [LINCS]		ACC 107	
Provider Batch ID	Vendor/Provider Batch ID number; Batch or lot number assigned to the cell line by the vendor or provider [LINCS]		No information	
Organism	Organism of origin; a controlled vocabulary describing the organism from which the cell line was derived (e.g. Homo sapiens, Mus musculus, etc.) [LINCS]		Human (Homo sapiens)	
Organ	Organ of origin; controlled terms describing the organ from which cell line is derived; (e.g. lung, mammary gland etc.) [LINCS]		Lung	
Tissue	Tissue of origin; A controlled vocabulary describing the tissue from which the cell line was derived [LINCS]		Lung carcinoma	

Cell type	A controlled vocabulary describing the cell type from which a cell line was derived, e.g. epithelial like, fibroblast-like, lymphoblast like, hematopoietic, mesenchymal, neural, etc. This provides information about cell morphology. Also sometimes referred to as cell morphology [LINCS]	epithelial
Growth properties	A controlled vocabulary describing the growth properties of the cell line (e.g. adherent, suspension) [LINCS]	growing adherently as monolayer
Biosafety Level		1
Donor sex	Describes sex of the organism from which the cell was obtained [LINCS]	man
Donor age	The age of the donor [LINCS]	58
Donor ethnicity	For human cells, the ethnicity of the donor [LINCS]	Caucasian
Donor health status	Controlled vocabulary describing the health status of the donor [LINCS]	No information
Disease	If the cell line came from a particular diseased tissue, the disease should be noted in terms of a controlled vocabulary (e.g. breast cancer, colon cancer, not diseased, etc.) [LINCS]	Lung tumor
Disease details	Additional description of a disease related to the cell line that may not be available in the disease ontology above [LINCS]	No information

Known mutations	Mutations inherent (from the donor) in the cell line, captured explicitly; e.g. if reference is not available [LINCS]	see citations
Mutation citations	Mutations inherent in certain cell lines; from a reference [LINCS]	DOI: 10.1093/jnci/51.5.1417; DOI: 10.1002/ijc.2910170110
Molecular features	Relevant molecular and morphological features of the Cell Line [LINCS]	Foster, KA; et al. (15 September 1998). "Characterization of the A549 cell line as a type II pulmonary epithelial cell model for drug metabolism"
Genetic modification	This field specifies any stable constructs as well as any genetic modifications (mutations, translocations) introduced into this cell line (e.g. H2B-mCherry integrated at the AAVS1 Safe Harbor locus). Details of the procedures used to generate this line (e.g. CRISPR/Cas9-mediated transformation) should be described and appropriate citations provided in the Production Details field [LINCS]	No information
Recommended culture conditions	A description of the standard tissue culture conditions (media, supplements, culture dish treatment) used to maintain the cell line. Description of culture dish treatment conditions would include information about coating of culture dish with fibronectin, collagen, etc., prior to cell plating. If special culture vessels are required to	at 37 °C with 5% CO_2 (depending on the used cell culture media)

	grow the cells, these should also be mentioned, and details provided [LINCS]	
Related projects	Other projects in which the cell line has been studied / used; A controlled vocabulary describing other large-scale projects in which the cell line has been used (e.g. ENCODE, TCGA, ICBP, Epigenomics, etc.) [LINCS]	A Multilaboratory Toxicological Assessment of a Panel of 10 Engineered Nanomaterials to Human Health—ENPRA Project—The Highlights, Limitations, and Current and Future Challenges, Kermanizadeh et al 2016
Verification reference profile	Expected STR (reference) profile of the cell line based on provider information, if available [LINCS]	Amelogenin: X,Y<br>CSF1PO: 10,12<br>D13S317: 11<br>D16S539: 11,12<br>D5S818: 11<br>D7S820: 8,11<br>TH01: 8,9.3<br>TPOX: 8,11<br>vWA: 14
Reference source	This field specifies a catalog entry or database record for this cell line at a cell type collection, if available, or an established external source [LINCS]	DOI: 10.1093/jnci/51.5.1417; DOI: 10.1002/ijc.2910170110
Cell markers	A controlled vocabulary describing the markers used to isolate / identify the cell type [LINCS]	None

Gonsome code	List of the sex chromosomes (gonosome) of the sample e.g. XX, XY, XXY [LINCS]	XY		
Disease site onset	Site of disease onset in primary cell donor [LINCS]	Cancer (lung carcinoma)		
Disease age onset	Age of disease onset in primary cell donor (in years) [LINCS]	58		
Donor disease duration	Age of death of primary cell donor (in years) [LINCS]	No information		
Cell relevant citations	List of references (with PMIDs) of relevance to cell line derivation, etc. [LINCS]	PubMed: 32094589; PubMed: 8709260; PubMed: 8642649; PubMed: 8642659; PubMed: 8662694; PubMed: 175022		
Culture conditions	A description of the culture conditions that were used and are suitable for this type of cell [LINCS]	at 37 °C with 5% CO2 with DMEM (Gibco #31885-023, Lot: 2183143 as cultivation medium and Gibco #11054-020, Lot: 2187119 as assay medium) + 10 vol% FBS (PAN Biotech)	Medium: DMEM w 1 g/ l glucose and 3,7 g/ l NHCO3 + 10 vol% FBS à requires 9 vol% CO2 atmosphere in the 37°C incubator; Seed with 1 to 1,5E+06 cells/ 75 cm2 , medium change every third day, subcultivation at 70-90% confluence; trypsination for 5 Min.	At 37 °C with 5 % CO2 (Note: Used DMEM contains w: 4.5 g/L Glucose, w: stable Glutamine, w/o: Sodium pyruvate, w: 3.7 g/L NaHCO3 - PAN Biotech P04-04500) + 10 vol% FBS (PAN Biotech)

Cell culturing material	Detailed description of the source and providers of the materials (cell culture plastic device)	Microscope (OLYMPUS, IX70)<br>Neubauer (Assistant)<br>Multistep pipette (Brand)<br>Single channel pipette (Eppendorf)<br>Incubator (37oC, 5% CO2, humidified atmosphere) (Thermo, Hera cell 240)<br>Centrifuge<br>Vortexer (Heidolph)<br>Water bath 37° (GFL 1086)<br>Sterile hood (Kojair Tech Dy, No 1575220 and Thermo, MSC-Advantage)<br>Culture media DMEM (Gibco, Thermo #31885023, Lot: 2183143)<br>Culture media DMEM without phenol red (Gibco, Thermo #11054020, Lot:2187119)<br>Sterile PBS (Sigma #D8537, Lot: RNBJ3292)<br>Rotisolv HPLC gradient water (Roth #A511.2)<br>L-Glutamine (Sigma, #59202C, Lot: SLBZZ36l)<br>FCS (Sigma #F75221, Lot: 060M3396)	Flasks: Greiner #658175, Lot E200439W Plates: Greiner #655180, Lot E17023KC<br>Serological pipets: Greiner #606180, 607180<br>Pipet boy: Brand accu-jet<br>Pipets: Eppendorf Research plus 0,5-10 µl, 10-100 µl, 100-1000 µl<br>Pipet tips: 10 µl Greiner # 771261, 1250 µl Greiner #750261, 200 µl Biozym #VT0240<br>Tubes: Sarstedt #62554502, 62547254, Greiner # 616201<br>Incubator: New Brunswick Galaxy 170R, 37°C, 9% CO2,humified<br>centrifuge: Eppendorf 5810R, 5418<br>BSC: Thermo Maxisafe 2030i<br>Water bath: GFL<br>Vacuum pump: Integra	
Cell seeding details	The number of cells seeded, percentage confluency at the start of an experiment, and time between seeding and experimentation should be reported for cell culture experiments. For experiments involving multiple cell types (e.g. co-culture, tumour spheroids), the number of non-target (e.g. healthy) cells and the number of target (e.g. diseased) cells should be	10.000 cells per well in a 96 well plate format or 1:10 or 1:5 for maintenance in T75 flasks	1E+06 cells/ 75 cm2 for cultivation10,000 cells/well in 96 well plate format	seed out at ca. 1-2 x 10^6 cells/75 cm2; split confluent cultures 1:7 to 1:12 every 4-7 days using trypsin/EDTA RV I – 17.000 cells/well RV II – 22.000 cells/well RV III – 22.000 cells/well

	reported. Ideally, however, the number of cells for each cell type present should be reported. [MIRIBEL]			
Mycoplasma testing	Have the cell lines been tested for mycoplasma contamination? In this checklist, indicate on which page (or section and paragraph) the information can be found. [Nature]	Negative in DAPI, microbiological culture, RNA hybridization, PCR assays		
Cell line authentication	Have the cell lines been authenticated? If so, by which method? [Nature]	STR analysis - confirmed		
Passage number	The number of times, if any that the cells have been re-plated and allowed to grow back to confluency or to some maximum density if using suspension cultures. [LINCS]	P10, P11 and P13	P9, 11, 13 or 19	P11, P12 and 12

Cell growth	Description before and during the test performance [Drasler]	Depending on the type of NM and the concentration, no difference in cell growth. Cell growth / doubling times was described for ca. 40 hours in SOP RRT001.10. Cell confluence in flask before seeding cell counting and seeding in 95 well plate format 90 – 95 %. Cell confluence before treatment 70 – 90 %.	Confluence before splitting 70-90 %<br>Confluence before treatment 70-80 %	
Morphology	Description before and during the test performance [Drasler]	Epithelial cells, growing adherently as monolayer. Depending on the type of NM and the concentration, no difference in cell morphology except for cells treated with 10, 20, 40 and 80 μg/cm2 ZnO NM110. Starting from the concentration of 10 μg/cm2 the cell shape got rounder, smaller confluency seemed to be less	No change during cultivation	Epithelial cells, growing adherently as monolayer
Justification of biological model	If applicable, give brief summary and references for the prediction model; Consider the intended purpose of the prediction model [OECD GD 211]	Well established and studied cell line and to compare with other study outcomes, e.g. : "A Multilaboratory Toxicological Assessment of a Panel of 10 Engineered Nanomaterials to Human Health—ENPRA Project—The Highlights, Limitations, and Current and Future Challenges", Kermanizadeh et al 2016		

3 Endpoint and read out information

Parameter	Parameter description	Partner 1	Partner 2	Partner 3
Method(s)	Methods for analysis e.g. microscopy, optical observation, ELISA<br>Describe the response and its measurement [OECD GD 211]<br>Overview on analytical method(s) to assess test endpoint(s) [ToxTemp]	Optical observation by microscope of A549 cells to check for morphology and densitiy before and after treatment with the distinct NMs<br>Measuring of absorbance of the samples using a measure wavelength at 450 nm and a reference wavelength at 630 nm with plate reader MultiscanGo (Thermo)<br>The assay is based on the cleavage of tetrazolium salts to formazan by cellular enzymes. An expansion in the number of viable cells results in an increase in the overall activity of mitochondrial dehydrogenases, and the formation of formazan is directly proportional to the number of metabolic active cells in the culture	Cellular viability - WST-1 assay in A549 cells	Optical observation of cells by microscope to evaluate viability subjectively<br>Absorbance measurement of possibly metabolized WST-1 reagent with plate reader Tecan infinite 200Pro<br>WST-1 reagent is metabolized by viable cells<br>Concentration dependent decrease of measured absorbance after 24h treatment with toxic/harmful substances/less viable cells
Type of method	Quantitative or qualitative	Quantitative	Quantitative	Quantitative
Test method purpose	What is the claimed purpose and rationale for intended use of method, e.g. alternative to an existing method, screening, provision of novel information in regulatory decision-making, mechanistic information, adjunct test, replacement, etc.	Evaluation of LC50 values for regulatory decision making.<br>Measurement of cellular toxicity and viability of NMs. To eliminate possible interferences from NMs with the absorbance readings and/or with the WST-1 substrate, the standard procedure from the reagent provider has been adapted by Vietti et al. 2013.<br>Alternatives: LDH, MTT, celltiter glow, XTT, neutral red assay	The aim of the method is to assess nanomaterials' cytotoxicity using the WST-1 assay. WST-1 reagent is metabolized by viable cells. Measuring of absorbance with plate reader.	Well established screening method suitable for high throughput, replacement of animal tests, alternative to measure radioactive labeled nucleosides or 5-bromo-2'-deoxyuridine

Scientific principle of the method	Provide scientific rationale; Description of scientific principle; Biological/physiological basis and relevance; Mechanistic basis; Is anchor point an AOP? [OECD GD 211]<br>Features of the test system that reflect the in vivo tissue [ToxTemp]	The cell proliferation reagent WST-1 is designed to be used for spectrophotometric quantification of cell proliferation, growth, viability, and chemosensitivity in vitro. The assay is based on the cleavage of tetrazolium salts to formazan by mitochondrial dehydrogenases. The formation of formazan is directly proportional to the number of metabolic active cells in the culture and thus is used as information of viability.<br>Test system could reflect in vivo tissue better by using primary cells and test substances and concentrations which reflects actual and realistic burden.	The cell proliferation reagent WST-1 is designed to be used for the non-radioactive, spectrophotometric quantification of cell proliferation, growth, viability, and chemosensitivity in cell populations using the 96-well-plate format. The assay is based on the cleavage of tetrazolium salts to formazan by cellular enzymes. An expansion in the number of viable cells results in an increase in the overall activity of mitochondrial dehydrogenases, and the formation of formazan is directly proportional to the number of metabolic active cells in the culture.	The tetrazolium salts are cleaved to formazan by cellular enzymes (10). An expansion in the number of viable cells results in an increase in the overall activity of mitochondrial dehydrogenases in the sample. This augmentation in enzyme activity leads to an increase in the amount of formazan dye formed, which directly correlates to the number of metabolically active cells in the culture. Quantification of the formazan dye produced by metabolically active cells by a scanning multiwell spectrophotometer (ELISA reader).The absorbance of the dye solution is measured at appropriate wavelengths.
Test relevance		Evaluation of LC50 values.	Evaluation of LC50 values	Relevant for assessment of hazard potential of compounds or effects on cell proliferation
(Potential) linkage to AOPs	Link to KE (key event) or MIE (molecular initiating event)? Which AO might be modelled? If no AOP present, give link between mechanism the assay measures and resulting hazard endpoint [OECD GD 211]	activity of mitochondrial dehydrogenases is proportional to formation of formazan which is directly proportional to the number of metabolic active cells	we studied the activity of mitochondrial dehydrogenases, which are indicators of the metabolic activity of functional mitochondria	activity of mitochondrial dehydrogenases in lung epithelial cells as MIE might reflect effects of NM on lung tissue
Method uses	Known uses: used in different laboratories? [OECD GD 211]	Yes, used in other laboratories. Well establishes assay to evaluate cell viability or mitochondrial dehydrogenase activity from various compounds and even well established for NMs	The WST-1 assay is the most common test to assess the in vitro cytotoxicity of chemicals. (Scarcello 2020)	WST-1 assay is a standard test in many also industrial labs to analyze effects of various compounds on cell viability/proliferation
Applicability of test method	Potential solubility issues with the test system, and solutions proposed to address the issue [OECD GD 211]		Not considered	

Reagents	Add all reagents used in your experiment [MIFlowCyt]<br>For mixtures, report the composition [OECD GD 211]	Culture media DMEM (Gibco, Thermo #31885023)<br>Culture media DMEM without phenol red (Gibco, Thermo #11054020)<br>WST-1 Stock Solution (2-(4-iodophenyl)-3-(4-nitrophenyl)-5-(2,4-disulphophenyl)-2H-teterazolium, Roche Diagnostics, #11 644 807 001) - stored in 1mL aliquots at -20°C<br>Triton X-100, CAS 9002-93-1 (Sigma #T8787)<br>Sterile PBS (Sigma #D8537)<br>Rotisolv HPLC gradient water (Roth #A511.2)<br>L-Glutamine (Sigma, #59202C, LotSLBZZ36l)<br>FCS (Sigma #F75221, Lot 060M3396)	DMEM (Gibco 31885) + 10 vol% FBS (PAN Biotech), 0,05% Trypsin/ 0,02% EDTA, without Ca2+/ Mg2+ in PBS PAN P10-23100, DPBS without Ca2+/ Mg2+ (Gibco 141900)<br><br>Assay medium: DMEM<br>(- phenol red)<br>(- glutamine)<br>+1% L-glutamine<br>+10% FCS	Culture media DMEM (PANBiotech # P04-04500)<br>Culture media DMEM without phenol red (Gibco, Thermo #11054020)<br>WST-1 Stock Solution (2-(4-iodophenyl)-3-(4-nitrophenyl)-5-(2,4-disulphophenyl)-2H-teterazolium, Roche Diagnostics, #11 644 807 001)<br>Triton X-100, CAS 9002-93-1 (Sigma T8787)<br>Sterile PBS (Gibco #141900144)<br>Sterile destilled water<br>Fetal bovine serum, Sera Plus (PANBiotech # P30-3702)<br>Stable Glutamine (PANBiotech # P04-82050)
Kit	The kit used to perform this experiment, if applicable. [MIFlowCyt]	WST-1 Stock Solution (2-(4-iodophenyl)-3-(4-nitrophenyl)-5-(2,4-disulphophenyl)-2H-teterazolium, Roche Diagnostics, #11 644 807 001) - stored in 1mL aliquots at -20°C	WST-1 Roche Diagnostics	WST-1 (Roche Diagnostics, #11 644 807 001)
Endpoint(s) of the test method	Response here makes reference to any biological effect, process or activity that can be measured [OECD GD 211]	Metabolic activity of mitochondrial dehydrogenases proportional to metabolic active cells, cytotoxicity	The Cell Proliferation Reagent WST-1 is designed to be used for the non-radioactive, spectrophotometric quantification of cell proliferation, growth, viability, and chemosensitivity in cell population.	Proliferation, growth, viability, and chemosensitivity
Endpoint-specific controls / mechanistic control compounds (MCC)		Positive control with 0.5% Triton x-100 which is gives an information of absorbance with test substance and no metabolic active cells Background absorbance controls (only medium with NMs in corresponding concentrations without cells)	Positive controls (cells exposed to Triton-X-100)Untreated cellsMedium + NM as background absorbance controls - concentration corresponding to test samples	Positive control (untreated cells, only medium) Negative control (dead cells, treated with 0.5% Triton X-100) Background absorbance controls (only medium or medium with corresponding concentration of test compound respectively)

Experiment variables	Experiment variables are attributes that differ between samples within an experiment due to pre-existing differences in sample states or due to experimental manipulation of the samples (e.g., smoker versus non-smoker, IL-2 treatment versus no treatment, knockout versus wild-type, or variable number of transplanted stem cells). [MIFlowCyt]	Treated vs untreated cells with different types of NMs and different concentrations.<br>Density of cells (%) before treatment<br>Speed of Experimenter (time duration for different steps)<br>Experience experimenter	No variables between samples within an experiment. Sample states and preparation constant	Treated with NM in different concentrations vs. untreated<br>2 different NM
Parameter (Investigation)	Measured values e.g. viability, fluorescence<br>Measured characteristics e.g. IL-6 cytokine expression [MIFlowCyt]	Metabolic activity of mitochondrial dehydrogenases proportional to formation of formazan	Metabolic activity of mitochondrial dehydrogenases proportional to formation of formazan	Absorbance of WST-1 metabolite
Relation between assay-response and in vivo/biology/physiology	What is relation between assay-response and in vivo/biology/physiology? [OECD GD 211]<br>Features of the test system that reflect the in vivo tissue [ToxTemp]	Assay gives information about NM cytotoxicity in A549 cells. A549 cells are lung epithelial cells and a model to test in vitro toxicity from various compounds including NMs which might me inhaled.	Assay gives information about NM cytotoxicity after incubation of NMs in A549 cells.	Assay gives information about activity of mitochondrial dehydrogenases in cells thus reflecting increased/decreased proliferation of cells or influence of NM on cell viability
Robustness of the method	Reliability of the experimental results, Within-laboratory repeatability and reproducibility, Between laboratory transferability and reproducibility [OECD GD 211]	Within-laboratory repeatability depends on the experimenter and the passage number of the cells?	WST-1 test is a well-established and widely used method.	þ
Reference chemicals/chemical libraries, rationale for their selection and other available information	Are results for the reference chemicals (i.e. the "training set" chemicals used in the development and evaluation of the assay and its associated prediction model) are free and publicly available in some form. If available, what is rationale for their selection. [OECD GD 211]	A Multilaboratory Toxicological Assessment<br>of a Panel of 10 Engineered Nanomaterials to<br>Human Health—ENPRA Project—The Highlights,<br>Limitations, and Current and Future Challenges, Kermanizadeh et al 2016	ENPRA Project: A Multilaboratory Toxicological Assessment<br>of a Panel of 10 Engineered Nanomaterials to<br>Human Health—ENPRA Project—The Highlights,<br>Limitations, and Current and Future Challenges, (Kermanizadeh 2016)	
Metabolic capacity of the test system	Is test system metabolically competent? Addition of enzymatic fraction? [OECD GD 211]	Measurement of metabolic active cells<br>Test substance can be transformed	Measurement of metabolic active cells with the WST-1 assay	

Steps towards mechanistic validation			None	
Method randomization	If a method of randomization was used to determine how samples/animals were allocated to experimental groups and processed, describe it. For animal studies, include a statement about randomization even if no randomization was used. [Nature]		None / No animal experiments	
Blinding	If the investigator was blinded to the group allocation during the experiment and/or when assessing the outcome, state the extent of blinding.<br>For animal studies, include a statement about blinding even if no blinding was done. [Nature]	Experimenter where blinded in NM cytotoxicity outcome<br>No previous experience with A549 cells<br>No previous experience with WST-1 assay from one experimenter<br>No previous experience with sonication as described in SOP	No blinding was investigated.	Investigator was „blinded" in that way that they had no further information about the experiment except the SOP and should perform the experiment according to their own interpretation of the SOP, no information about the effect of the reference NM was provided
Acceptance criteria for source cell population	Acceptance criteria assessed at different stages of test system and method, furthermore assessing variability and troubleshooting. [ToxTemp]	Microscopical check for cell morphology and confluence before cell culturing and treatment and WST-1 assay<br><br>Result Person A:<br>1.)<br>2.)<br>3.) 90 - 95 % conflucence before splitting<br>Result Person B:<br>1.) 90 % confluence before splitting<br>2.) 100 % confluence before splitting<br>3.) 70 % confluence before splitting	Microscopic observation of cell morphology and viability during cultivation and before the NM incubation<br><br>Result Person A:<br>1.) 90 % confluence before splitting<br>2.) 90 % confluence before splitting<br>3.) 60 % confluence before splitting<br>Result Person B:<br>1.) 90 % confluence before splitting<br>2.) 80 % confluence before splitting<br>3.) 80 % confluence before splitting	Calculation of doubling time by cell counts during each subculturing procedure and monitored over time.<br>Testing of cell viability after trypsinization. A healthy culture should contain at least 80 % viable cells.

Acceptance criteria for assessing test system at its start		Morphology and confluence of cells<br><br>Result Person A:<br>1.)<br>2.) 80 - 85 % confluence before NM<br>3.) 80 - 85 % confluence before NM<br><br>Result Person B:1.) 70 % confluence before NM2.) 90 % confluence before NM3.) 90 % confluence before NM	70 % confluence of cells<br>Healthy culture should contain at least 80 % viable cells<br>Cell morphology by microscopy<br><br>Result Person A:<br>1.) 40-50 % confluence before NM<br>2.) 40-50 % confluence before NM<br>3.) 40-50 % confluence before NM<br><br>Result Person B:1.) 80 % confluence before NM2.) 70 % confluence before NM3.) 70-80 % confluence before NM	70% confluence of cells<br><br>Result Person A and B (no information):<br>1.) % confluence before NM<br>2.) % confluence before NM<br>3.) % confluence before NM
Acceptance criteria for the test system at the end of compound exposure	Criteria to accept or reject experimental data [OECD GD 211]	Absorbance at 450 nm should be between 0.5 and 2,- Cells exposed to the positive control should be metabolic inactive<br>Background absorbance controls (NMs without cells) should be included when testing with NMs<br>To avoid interference with WST-1 and NM´s better Medium without phenolred<br><br>Result Person A:<br>1.) Abs.(ctr.): 1,7-2,0 Max(SD): 0,3<br>2.) Abs.(ctr.): 1,6-2,1 (MW=1,8) Max(SD): 0,4<br>3.) Abs.(ctr.): 1,3-1,6 Max(SD): 0,1<br><br>Result Person B:<br>1.) Abs.(ctr.): 0,9-1,0 Max(SD): 0,2<br>2.) Abs.(ctr.): 1,2-1,5 Max(SD): 0,1<br>3.) Abs.(ctr.): 1,0-1,5 Max(SD): 0,2	A450 nm- A630 nm of medium controls (cells incubated in culture media without phenol red) should be between 0.5 and 2, standard deviation should be <0.3<br>Positive controls (cells exposed to Triton-X-100) should be lower than the controls<br><br>Result Person A:<br>1.) Abs.(ctr.): 0,4-0,5 Max(SD): 0,1<br>2.) Abs.(ctr.): 0,6-1,0 Max(SD): 0,2<br>3.) Abs.(ctr.): 0,7-0,9 Max(SD): 0,1<br><br>Result Person B:<br>1.) Abs.(ctr.): 1,2-1,3 Max(SD): 0,2<br>2.) Abs.(ctr.): 1,1-1,2 Max(SD): 0,1<br>3.) Abs.(ctr.): 1,0-1,2 Max(SD): 0,1	Absorbance at 450 nm - absorbance at 630nm of:<br>- A450 nm- A630 nm of medium controls (cells incubated in culture media without phenol red) should be between 0.5 and 2, standard deviation should be <0.3.<br>- Positive controls (cells exposed to Triton-X-100) should be lower than the control<br><br>Result Person A:<br>1.) Abs.(ctr.): 1,8-2,2 (MW=2,0) Max(SD): 0,15<br>2.) Abs.(ctr.): 1,5-1,8 Max(SD): 0,1<br>3.) Abs.(ctr.): 3,0-3,2 Max(SD): 0,07<br><br>Result Person B:<br>1.) Abs.(ctr.): 2,3-2,4 Max(SD): 0,11<br>2.) Abs.(ctr.): 1,9-2,0 Max(SD): 0,21<br>3.) Abs.(ctr.): 2,3-2,5 Max(SD): 0,09
Raw data format		.txt<br>.xlsx	.xls	.xlsx
Internal data storage		xx	xx	xx

Experimental system(s) used	Experimental test system extensively documented at different stages. [ToxTemp]		Microscopic observation - pictures available	
Equipment used, calibration program		Microscope (OLYMPUS, IX70) Neubauer (Assistant) Multistep pipette (Brand) Single channel pipette (Eppendorf) Incubator (37oC, 5% CO2, humidified atmosphere) (Thermo, Hera cell 240) Centrifuge Scanning multiwell spectrophotometer with filters for 450 nm and a reference filter for 630nm (Thermo, Multiscan GO) Vortexer (Heidolph) Water bath 37° (GFL 1086) Sterile hood (Kojair Tech Dy, No 1575220 and Thermo, MSC-Advantage)		Microscope: Zeiss Axio Vert A1 w/ LD A-Plan 10x Hemacytometer: Nano EnTek C-Chip Neubauer improved, DHC-N01 Multistep Pipette: Eppendorf Multipette E3x Pipette: Eppendorf Research 5000, Reference 100, 200, 1000, 20, 10 Incubator: Binder 9040-0112, 37°C 5% CO2, humidified atmosphere Centrifuge: VWR Mega Star 1.6R w/ rotor 3659 Spectrophotometer: Tecan Infinite M200Pro w/ Tecan i-control Version 2.0.10.0 Sonicator: Bender & Hobein Laboson 200 Ultrasound bath Sterile hood: Thermo Scientific MSC-Advantage Vortexer: Heidolph REAX 1 R w/ 50Hz Waterbath: Memmert at 37°C
Availability of internal standards (e.g. positive and negative controls)		Triton x-100 as positive control for metabolic inactive cells as all dead<br>Various other compounds also positive controls (E.g. specific NMs based on previous experiments)<br>Negative control: No treatment with compounds	Positive control: Triton X-100<br>Negative control: cells incubated with media	Not available in first cycle<br>Available in repeated experiments: Triton X-100 as positive control, untreated as negative control
Limit of detection and limit of quantification, detection range		Absorbance values >=4.0	A450 nm- A630 nm of medium controls (cells incubated in culture media without phenol red) should be between 0.5 and 2, standard deviation should be <0.3<br>Positive controls (cells exposed to Triton-X-100) should be lower than the controls	Absorbance values >3.5 resulted in “Over” values

Cross-reference to related test methods	Related assays if known that may characterize the same key event [OECD GD 211]	MTT, LDH, CellTiter Blue Assay	Tetrazolium Reduction Cell Viability Assays<br>Resazurin Reduction Cell Viability Assay<br>Dead-cell Protease Release Cytotoxicity Assay<br>Lactate Dehydrogenase (LDH) Release Cytotoxicity Assays	Cell viability, chemosensitivity: CellTiter Blue Assay, ATP assay, LDH leakage and others
Additional quality control experiments	If another experiment was performed for the purposes of establishing quality control standards, that experiment may be referenced. [MIFlowCyt]	WST-1 assay in combination with other assay like LDH can give information if test compound is just inhibiting mitochondrial dehydrogenases or if compound is cytotoxic and cell viability is decreased	None	
Dispersion protocol	Careful consideration of the dispersion protocol effects for NMs: e.g. avoidance of ROS generation and other effects on NM; avoidance of stabilizers with toxic or mutagenic potential. Tested for presence of endotoxins. [Drasler]	According to NanoS-QM SOPs "Preparation of nanoparticle dispersions from powdered material using ultrasonic disruption" and "Probe sonicator calibration" Due to technical limitations it was not possible to sonicate with 7.35±0.05 Watt. Therefore sonication time was calculated to sonication time of 13 min 50 sec was calculated to deliver a total acoustic energy of 7056±103 J.<br><br>NM weight + Volume<br>Result Person A:<br>1.) NM110: 19.04 + 7,44, NM101: 20.77 + 8.11<br>2.) NM110: 19.64 + 7,67, NM101: 26.59 + 10.39<br>3.) NM110: 19.41 + 7.58, NM101: 18.99 + 7.42	According to NanoS-QM SOPs "Preparation of nanoparticle dispersions from powdered material using ultrasonic disruption" and "Probe sonicator calibration"Sonication set up for placement of sonication vial in ice-water bath needs to be adjusted. To insert the sonicator probe one third into the dispersion we need to prepare higher sample volume.<br><br>NM weight + Volume<br>Result Person A:<br>1.) 79,7 mg TiO2/ 31,1 ml und 70,7 mg ZnO/ 27,2 ml<br>2.) 75,7 mg TiO2/ 29,1 ml und 65,2 mg ZnO/ 25,1 ml<br>3.) 61,6 mgTiO2/ 23,7 ml und 75,5 mg ZnO/ 29 ml	According to NanoS-QM SOPs "Preparation of nanoparticle dispersions from powdered material using ultrasonic disruption" and "Probe sonicator calibration" Variation from SOP: no sonicator with probe sonotrode was used but an ultrasonic bath. Temperature for calibration was measured in a beaker according to SOP 1 cm next to the beaker wall. A sonication time of 10 min 26 sec was calculated to deliver a total acoustic energy of 7056±103 J.<br><br>NM weight + Volume<br>Result Person A:<br>1.) NM110: 20,88 + 8,156, NM101: 24,79 + 9,684<br>2.) NM110: 20,88 + 8,156, NM101: 24,79 + 9,684<br>3.) NM110: 24,55 + 9,990, NM101: 32,24 + 12,594

		NM weight + Volume<br>Result Person B:<br>1.) NM110: 18.95 + 7.4, NM101: 23.34 + 9.12<br>2.)<br>3.) NM110: 18.02 + 7.039, NM101: 17.47 + 6.824	NM weight + Volume<br>Result Person B:<br>1.)<br>2.)<br>3.)	NM weight + Volume<br>Result Person B:<br>1.) NM110: 17,56 + 6,85, NM101: 34,97 + 13,66<br>2.) NM110: 17,56 + 6,85, NM101: 34,97 + 13,66<br>3.) NM110: 32,79 + 12,8, NM101: 21,76 + 8,5
Dispersing agent		HPLC H20 + 2% FCS	MiliQ water + 2 % FCS	DEPC Water + 2% FCS
Dispersing handling	Descirption of the dilution technique	Person A:NM suspension dilutions were prepared as follows: 1/10 dilution of the NM stock with cell culture media. For the dilution series, 18ml assay medium with 2ml HPLC / 2% FCS were prepared and placed 1ml each in 2ml Eppis. 2-fold serial dilutions of the materials, with the highest dose of 80 µg/cm2 were prepared by pipetting 1 mL of the NM solution into the 2 mL Eppendorf tube. The solutions were mixed by vortexing.	Person A: NM wurden mit dem berechneten Volumen Dispersionsmedium angesetzt und nach Homogenisieren 1:10 mit Medium verdünnt (200 µl SL + 1800 µl Medium). daraus wurden die weiteren Verdünnungen laut Pipettierschema in der SOP wie folgt angesetzt: 1000 µl Dispersion medium wurden in 2 ml Reaktionsgefäß mit angehängtem Schnappdeckel (Eppi) vorgelegt. Es wurden 1000 µl SL dazu pipettiert. Das Gefäß wurde ca. 20 s mit geschlossenem Deckel gevortext und anschließend kurz abzentrifugiert. Es wwurden 1000 µl entnommen und ins nächste Eppi transferiert, das bereits 1000 µl Dispersionsmedium enthielt. Usw.	

		Person B:<br>NM suspension dilutions were prepared as follows: 1 mL of the original NM stock was diluted with 9 mL cell culture media. 2 mL Eppendorf were used for further dilution steps. 1 mL cell culture medium diluted with HPLC + 2% FCS was prepared in 2 mL Eppendorft tubes. 2-fold serial dilutions of the materials, with the highest dose of 80 µg/cm2 were prepared by pipetting 1 mL of the NM solution into the Eppendorf tube with 1 ml of diluted cell culture media. The solutions were mixes by pipetting 10 times the volume up and down.	Person B:<br>NM suspensions were prepared as follows: A 24-Well plate was used for the suspensions. 0 µL (first well) and 1000 µL (8 wells) of the dispersion media [Dispersion media without NMs for the NM dilution steps (dilute HPLC water supplemented with 2% FCS 1:10 in full cell culture media, e.g. 9 ml cell culture media + 1 ml HPLC with 2%FCS)] was applied into the wells of the 24 well plate. Starting with 2000 µL of the Stock suspension (256 µg / mL) (first well), 1000 µL from each well was transferred into 1000 µL of dispersion media. Mean 1000 µL (1/2 of the initial volume) was transferred each time. The solutions were mixed by three times pipetting the volume up and down after each transfer.	
Time until NM application		Result Person A:1.) NM110 54 min, NM101 75 min2.) NM110 52 min, NM101 74 min3.) NM110 60 min, NM101 68 min<br><br>Result Person B:<br>1.) 20 min<br>2.) 20 min<br>3.) 20 min<br>Time after sonication for preparation of particle concentrations and application	Result Person A:1.) 2.) 3.)<br><br>Result Person B:<br>1.) 56 min<br>2.) 48 min<br>3.) 50 min<br>Time for preparation of both NM by sonication, preparation of particle concentrations and application	Result Person A: 20 Min<br><br>Result Person B:<br>30 Min
Particle concentrations		256, 128, 64, 32, 16, 8, 4, 2, 1 µg/ml	256, 128, 64, 32, 16, 8, 4, 2, 1 µg/ml	256 ug/ml, 128 ug/ml, 64 ug/ml, 32 ug/ml, 16 ug/ml, 8 ug/ml, 4 ug/ml, 2 ug/ml, 1 ug/ml, 0 ug/mL

Height of medium on the top of the cell layer		100 µl / Well in 96 well plates with 0,32 cm^2	100 µL / Well with 0,32 cm2	100 ul
Perfusion with medium			static incubation	
Protein corona	The effects of the protein corona formation should be considered, if possible. [Drasler]		Not considered	
Consideration of the concentration	High doses are required in mechanistic studies, however it needs to be considered if the concentration of MNs in tests would exceed the level at which agglomeration is enhanced. [Drasler]	High dose and low doses tested	Concentration depending agglomeration were investgated by IWT	
Units: mass or number concentration per cell	however it is recommended that all the three mass metrics are reported (mass, surface area and number) [Drasler]	Cell densitiy/confluence is known as 10.000 cells per Well are incubated. Proliferation time of A549 cells in distinct medium is known and surface are of 96 plate well	Not considered	
Intracellular NM concentration	In the likelihood of intracellular NM alterations or dissolution, detection of intracellular NM concentration is recommended. [Drasler]	Not known	Not considered	
Deposited dose on the cell surface	Deposited dose on the cell surface needs to be calculated (specified methods or models). [Drasler]	80 µg/cm2, 40 µg/cm2, 20 µg/cm2, 10 µg/cm2, 5 µg/cm2, 2,5 µg/cm2, 1,25 µg/cm2, 0,625 µg/cm2, 0,3125 µg/cm2	80 ug/cm2, 40 ug/cm2, 20 ug/cm2, 10 ug/cm2, 5 ug/cm2, 2,5 ug/cm2, 1,25 ug/cm2, 0,625 ug/cm2, 0,3125 ug/cm2	80 ug/cm2, 40 ug/cm2, 20 ug/cm2, 10 ug/cm2, 5 ug/cm2, 2,5 ug/cm2, 1,25 ug/cm2, 0,625 ug/cm2, 0,3125 ug/cm2
Submerged test conditions	Submerged test conditions: 1–100 µg MNs/mL, with the lower and higher limits at 0.125 and 200 µg/mL. It is recommended that the concentrations used in the submerged settings do not exceed greatly the level at which agglomeration is enhanced. [Drasler]	All concentrations are (0.3125 – 256 µg/mL)	Concentration depending agglomeration were investgated by IWT	

Dose selection	Doses selected should be anchored by known human exposures; conversions from human exposures<-> in vitro exposures are suggested (via e.g. reverse dosimetry models). [Drasler]	Dose selection based on ENPRA project and on establishes concentrations for NMs to evaluate LC50 values	Dose selection based on ENPRA project	
Dosimetric conversions	Use of specific model systems<br>e.g. Air-liquid interface (ALI) protocols for lung cell experiments may overcome the issue with suspension cultures by a direct deposition of a NM onto the lung cell surface. Use of ALI (and barrier protocols) avoids the need for many dosimetric conversions since most of the aerosolization<br>systems can measure the deposited NM concentration on-line thus allowing to obtain a dose-related effect (Braakhuis et al., 2016) [Drasler]		No conversions made	
Parameter / Endpoints	Description of the measured parameter<br>Multiple assays for individual endpoints should be employed. [Drasler]<br>Toxicological hazard endpoint being addressed [OECD GD 211]	Metabolic activity of A549 cells after treatment with different types and different concentrations of NMs	Metabolic activity of A549 cells after incubation with different NM concentrations	Endpoint addressed: ability of A549 cells to metabolize tetrazolium salts to formazan, cell viability, cell proliferation
(NM) interferences	Possible NM interferences with assay reagents, products or optical pathways need to be considered. [Drasler]	Durchführung INM	Determination of optical activities for all used NM dispersions / concentrations<br>Interferences: NM with impurities or ions of the dispersing agent<br>- No test adaptions	Durchführung INM<br>NM110 – interference with the assay reagent in the highest concentration<br>NM101 – interference with the assay reagent in the highest concentration
Sample size calculation	How was the sample size chosen to ensure adequate power to detect a pre-specified effect size? [Nature]	N=3 experiments and n=4 wells per test concentration and n=2 wells per test concentration and positive control and absorbance control	In accordance to ENPRA project	

Controls (reference material, positive and negative)	Chemical positive and negative controls need to be included in each assay and the use of NM-based negative and positive controls is recommended. [Drasler]	positive control: Triton X-100 negative control: cells incubated with media	Positive control: Triton X-100Negative control: cells incubated with media	Negative control: Triton X-100 Positive Control: None except untreated condition
Exposure	e.g. aerosol, dispersion	Dispersion	Dispersion	Dispersion
Dose / Concentration	administered dose, the initially added dose	256, 128, 64, 32, 16, 8, 4, 2 and 1 µg/ml	256, 128, 64, 32, 16, 8, 4, 2 and 1 µg/ml	256 ug/ml, 128 ug/ml, 64 ug/ml, 32 ug/ml, 16 ug/ml, 8 ug/ml, 4 ug/ml, 2 ug/ml, 1 ug/ml, 0 ug/mL
Concentration settings	Number of doses/concentrations, testing range	10 doses including control	10 NM concentrations / controls	10 concentrations including controls
Number of replicates		4 replicates/concentration, 2 replicates/ positive control (Triton-X100), 2 replicates/concentration as a blank	4 replicates/concentration, 2 replicates/ positive control	4 replicates/concentration, 2 replicates/ positive control
**Cells:**		A549 cells	A549 cells	A549 cells
Cellular model system	NM response need to be assessed in multiple (at least three) different cell types and/or co-cultures. [Drasler]	Human lung epithelial cells	Human lung epithelial cells	Lung epithelial cells
Method(s)	Description of the biological model system	Described in NanoS-QM: Standard operating procedure (SOP)<br>SOP-RRT001-1.0: Culturing A549 cells	Cells were seeded in a 96-well plate growing as adherent as monolayer	Probe sonicator calibration by the measurement of the temperature increase.<br>Cultivation of A 549 in 96-well plate<br>Preparation of NM suspensions<br>Treatment of cells with NM treated and readout with WST-1 reagent in Tecan
Culture dimensions	e.g. 2D/3D, spheroids or monolayer	2 dimensional	2D	2dimensional epithelial like
Toxicity/viability studies		Many, based on round robin ENPRA project	Part of the investigation / round robin test - see control samples	

Signal of cells with nanomaterial		Depending on concentration and type on NMs different metabolic activity	Interference: Intrinsic optical activity increase with concentration of applied NM. - No test adaptions<br>- Test SOP provides a blank of the nanomaterials at each concentration. The results are subtracted as a sample blank at each measurement.	
